# Abortive and productive infection of CNS cell types following in vivo delivery of VSV

**DOI:** 10.1073/pnas.2406421121

**Published:** 2024-08-19

**Authors:** Tyler B Krause, Constance L. Cepko

**Affiliations:** ^a^Department of Genetics, Blavatnik Institute, Harvard Medical School, Boston, MA 02115; ^b^Howard Hughes Medical Institute, Chevy Chase, MD 20815

**Keywords:** vesicular stomatitis virus (VSV), abortive infection, interferon, central nervous system (CNS)

## Abstract

Viral infection is frequently assayed by ongoing expression of viral genes. These assays fail to identify cells that have been exposed to the virus but limit or inhibit viral replication. To address this limitation, we used a dual-labeling vesicular stomatitis virus (DL-VSV), which has a deletion of the viral glycoprotein gene, to allow evaluation of primary infection outcomes. This virus encodes Cre, which can stably mark any cell with even a minimal level of viral gene expression. Additionally, the virus encodes GFP, which distinguishes cells with higher levels of viral gene expression, typically due to genome replication. Stereotactic injections of DL-VSV into the murine brain showed that different cell types had very different responses to the virus. Almost all neurons hosted high levels of viral gene expression, while glial cells varied in their responses. Astrocytes (Sox9+) were predominantly productively infected, while oligodendrocytes (Sox10+) were largely abortively infected. Microglial cells (Iba1+) were primarily uninfected. Furthermore, we monitored the early innate immune response to viral infection and identified unique patterns of interferon (IFN) induction. Shortly after infection, microglia were the main producers of *IFNb*, whereas later, oligodendrocytes were the main producers. *IFNb*+ cells were primarily abortively infected regardless of cell type. Last, we investigated whether IFN signaling had any impact on the outcome of primary infection and did not observe significant changes, suggesting that intrinsic factors are likely responsible for determining the outcome of primary infection.

## Significance

Virus–host interactions can vary widely, with some host cell types resisting virus entry or replication, and others supporting a full viral replication cycle. Such interactions have not been easy to characterize in vivo, particularly for infections of the central nervous system (CNS). We developed a dual-labeling virus with the capability of distinguishing abortive or productive viral replication in vivo. We used this virus to examine the response of different CNS cell types to infection with vesicular stomatitis virus. Additionally, we were able to uncover early host responses and assess their impact on primary infection. Investigation of other virus–host interactions can use this dual labeling strategy to better track the dynamics and specificity of virus–host interactions.

Neurovirology is a burgeoning field due to the clinical relevance of neurotropic viruses and the use of viruses as agents of gene delivery to the nervous system (1–3). Despite this interest, basic questions remain, due to the complexity of the nervous system and the difficulty in accurately representing virus–host interactions in vitro. A convergence of emerging technologies and recent discoveries allow us to investigate virus–host relationships in vivo to gain a greater understanding of these relationships.

A fundamental aspect of virus–host interactions is the viral tropism. The tropism is gated by the cellular expression of a virus’ receptor and a cell’s response, which can restrict viral infection/replication. These initial stages are not usually appreciated in studies of viral infection, which often use replication outcomes as the assay of infection. In addition, there are conflicting data in the literature, likely due to differences in virus species and strain, as well as the infection timing, location, and system of infection (i.e. in vitro vs in vivo), all of which can significantly influence outcomes (4, 5). Furthermore, this complexity is compounded by the more recent recognition of previously underestimated intermediate states of infection (6–8).

Conventional methods for monitoring tropism often involve binary readouts, distinguishing between infected and uninfected cells. Alternatively, studies that aim to quantify more graded responses use the titer of infectious virus produced by a tissue, e.g. a brain region. We now have newer techniques that enable an understanding of different stages of viral infection, including those where the virus is present, but unable to actively replicate, referred to as abortive infection. For example, rabies virus (RABV) can abortively infect the brain, where abortive infection is correlated with expression of IFNb, a constituent of the Type I Interferon (IFN) response, and a vital component of the innate immune system (8, 9). This observation supports a more recent hypothesis that suggests infection infidelities are one of the main factors driving innate immunity (10).

We sought to determine the response of different cell types in the brain to viral infection in vivo, using a virus that enables clear read-outs of different outcomes. Vesicular stomatitis virus (VSV), a well-characterized, nonsegmented, negative-strand RNA virus, was used. VSV has been used as a neurosynaptic tracer in our laboratory, where we have encountered low efficiency of transmission when attempting to use it as a monosynaptic tracer (11). As VSV has been characterized as sensitive to Type 1 interferon, primarily in studies carried out in vitro or in nonbrain tissues, we also were interested in the Type 1 response to VSV in the brain (12–14). In addition to its use as a neurosynaptic tracer, VSV has been used in the brain as an oncolytic agent for glioblastoma, and it is being used to deliver vaccines (15–18). A greater understanding of the response of different host cell types in the brain was thus of interest. To track infection and the response of different types of cells in vivo, we modified VSV to encode Cre, as a sensitive indicator of low-level viral transcription, as occurs in abortive infection, as well as GFP, as a reporter of viral replication. Using sensitive fluorescent in situ hybridization (FISH) methods and immunohistochemical markers of CNS cell types, the infection status of different cell types was quantified over time. The FISH method also allowed us to track expression of Type 1 IFN and interferon-stimulated genes (ISGs) and relate these to the infection status of different cell types. In addition, using mice defective for specific IFN responses, we were able to determine the role of this cytokine in early viral infection. These data provide a few surprises regarding the responses of different CNS cell types over time, with the production of IFNb changing from the predicted microglial cell type to that of oligodendrocytes.

## Results

### Dual Labeling VSV (DL-VSV) Has Host and Viral Readouts of Infection.

**Fig. 1.**
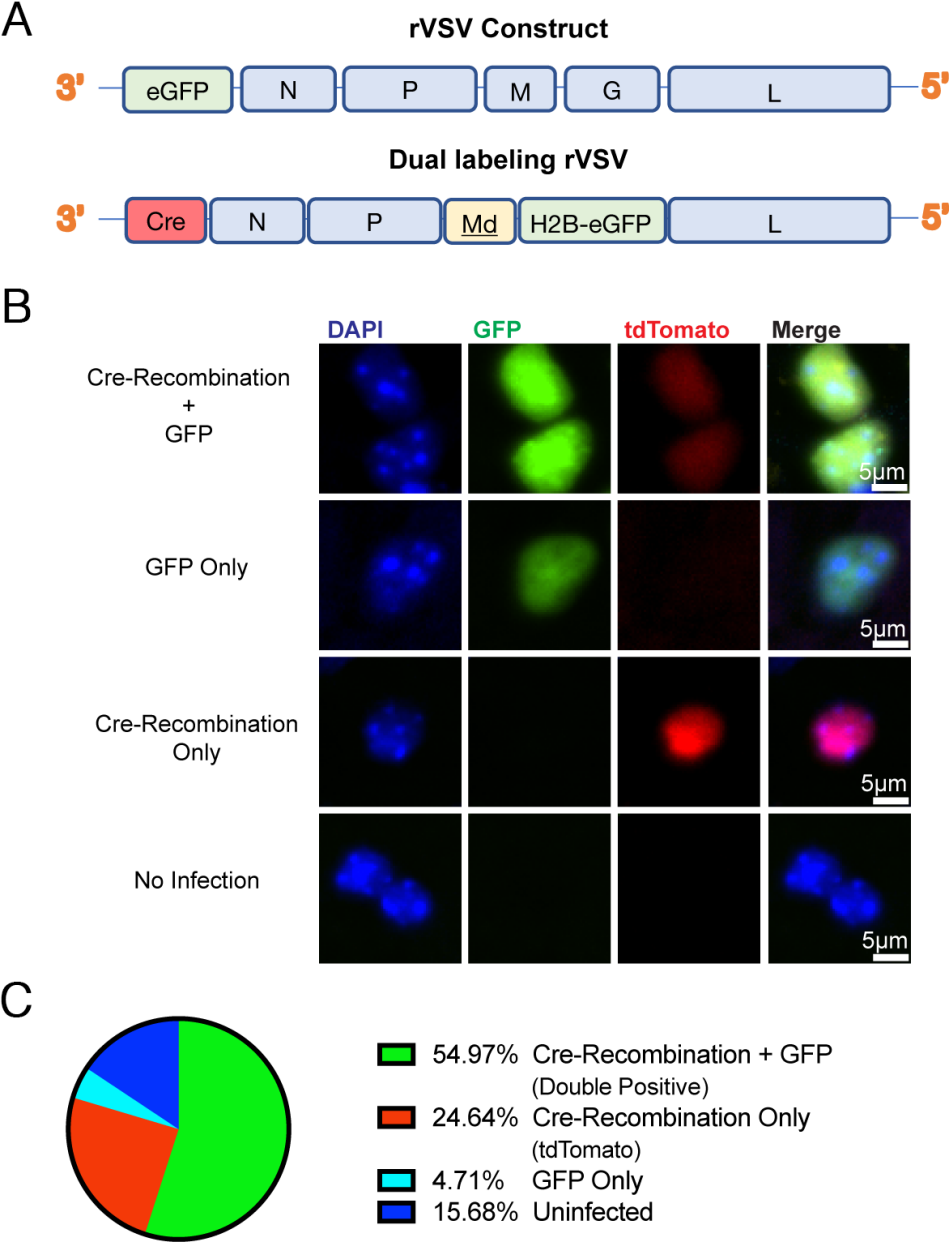
DL-VSV design and infection outcomes. (*A*) Diagram of traditional rVSV genome and the DL-VSV design. (*B*) Representative single-cell examples of differential infection status based on infection markers. (*C*) Percentage of infected region classified by infection outcomes. Average taken from ten brain sections.

To capture both host and viral readouts of infection, we made several modifications to a VSV genome that we had previously designed as a transsynaptic tracer virus (Fig. 1*A*). These modifications included removing the glycoprotein gene (G) and swapping in a nuclear-localized GFP (H2B-eGFP), introducing a Cre recombinase gene, and mutating the gene encoding the matrix (M) protein to make it less cytopathic (19). These modifications created a replication-defective virus that enables stable labeling of cells from primary transcription of Cre, which can lead to recombination of a conditional allele of a gene encoding a fluorescent protein, present in the mouse genome. Cells that support viral replication can express H2B-eGFP, whose accumulation is dependent upon secondary transcription following replication of the viral genome. To test whether the DL-VSV can induce Cre-recombination in the absence of viral replication, we made mutants that lacked the nucleocapsid (N), phosphoprotein (P) genes. In the absence of these genes, VSV cannot replicate (20). When infecting 293 T cells with this crippled VSV, and including N, P, L, and a Cre reporter plasmid in trans, Cre-recombination (tdTomato) and GFP expression were observed (*SI Appendix*, Fig. S1). When N, P, and L were not supplied in trans, tdTomato, but not GFP, was observed.

It has been reported that Cre mRNA can be transferred via extracellular vesicles to bystander cells where it can lead to recombination (21). We thus investigated whether Cre-recombination occurred in noninfected cells in the in vitro assay system. When either the lysate of infected cells, or intact infected cells, were transferred onto a monolayer of uninfected 293 T cells transfected with a Cre reporter plasmid, no Cre-recombination was observed in noninfected cells (*SI Appendix*, Fig. S2*B*).

We examined whether differential infection readouts would occur in vivo upon DL-VSV infection. DL-VSV was injected into the striatum of transgenic mice that possess nuclear-localized tdTomato upon Cre-recombination (Ai75d mice). We collected and imaged the brains 16 h postinfection (hpi). Several infection phenotypes, based on the presence or absence of the infection markers, were seen (Fig. 1*B*). Within an infected area, approximately 55% of cells were positive for both Cre-recombination (tdTomato) and GFP, while ≈25% of the cells displayed only Cre-recombination (Fig. 1*C*). Additionally, ≈5% of cells expressed only GFP and ≈16% did not exhibit any infection markers.

**Fig. 2.**
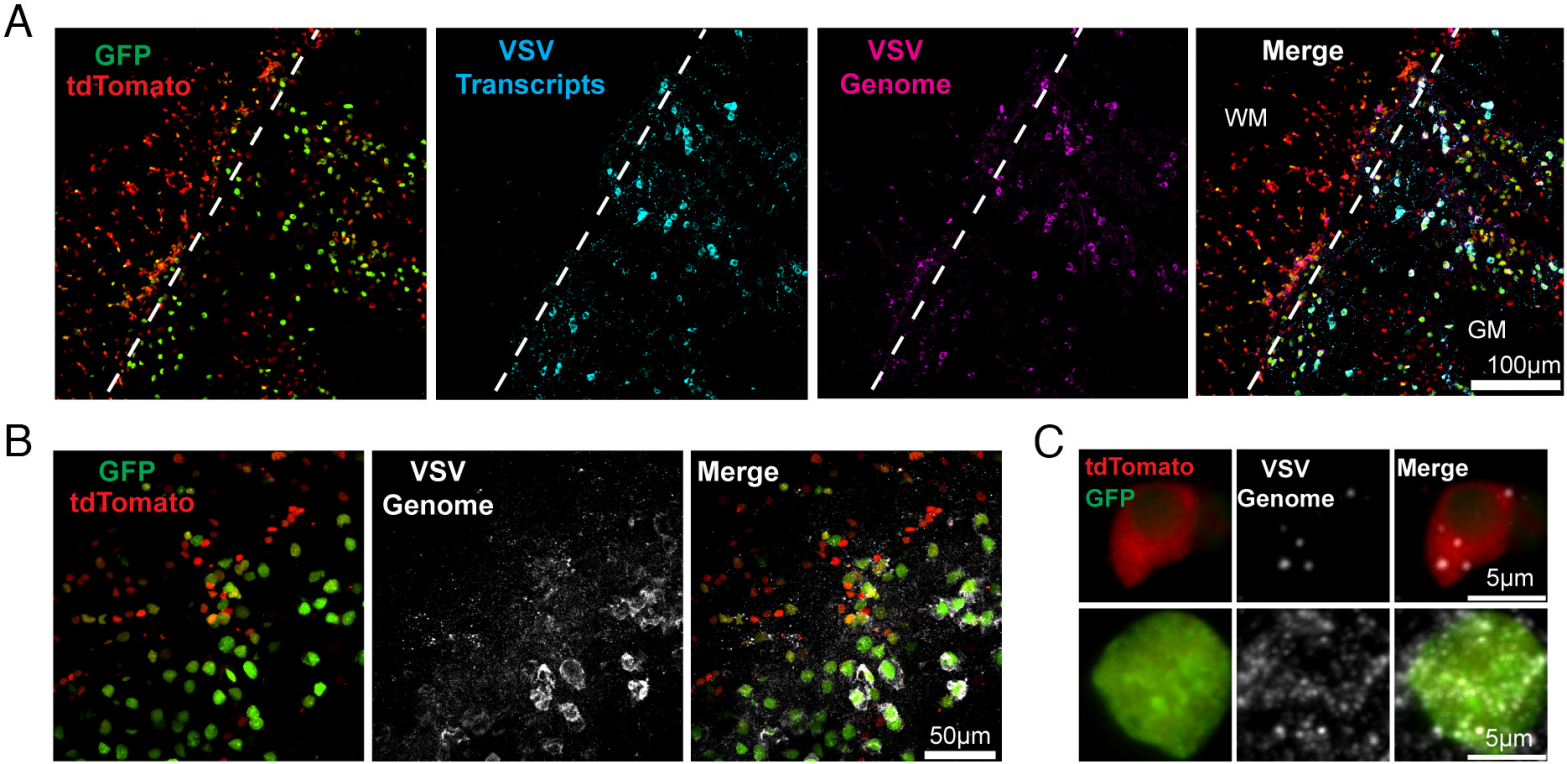
VSV genome and transcripts/anti-genome in the infected region. (*A*) 20× image of DL-VSV infected region depicting infection markers, VSV transcripts (cyan), and VSV genomes (magenta). Infected region spans white matter (WM) left of the dotted line and gray matter (GM) right of the dotted line. (*B*) 60× image of infected region and VSV genomes (white). (*C*) Magnified insets of images from B depicting single cells with infection markers and VSV genomes. All images were taken 16 h postinfection.

To determine whether the infection markers reflected the infection state in vivo, FISH experiments were carried out. Probes that could distinguish viral genomes versus viral transcripts/anti-genomes were used. Anti-genomes should only be present when the genome was replicated, and transcripts should be abundant following genome replication. VSV transcripts were seen in regions with more GFP+ cells. VSV genomes were detected in regions with tdTomato-only cells, as well as in regions with GFP+ cells (Fig. 2 *A* and *B*). At the individual cell level, viral genomes could be detected in cells exhibiting only tdTomato(Fig. 2*C*). In GFP+ cells, FISH signal for transcripts/viral genomes was much higher than in tdTomato-only cells(Fig. 2 *A* and *C*). These findings suggest that cells exhibiting GFP have undergone viral replication and are thus producing a high level of viral genomes and transcripts, while cells that express only tdTomato were abortively infected, i.e., had limited or no viral replication.

### Characterization of Infection Status of Different Cell Types Using DL-VSV In Vivo.

**Fig. 3.**
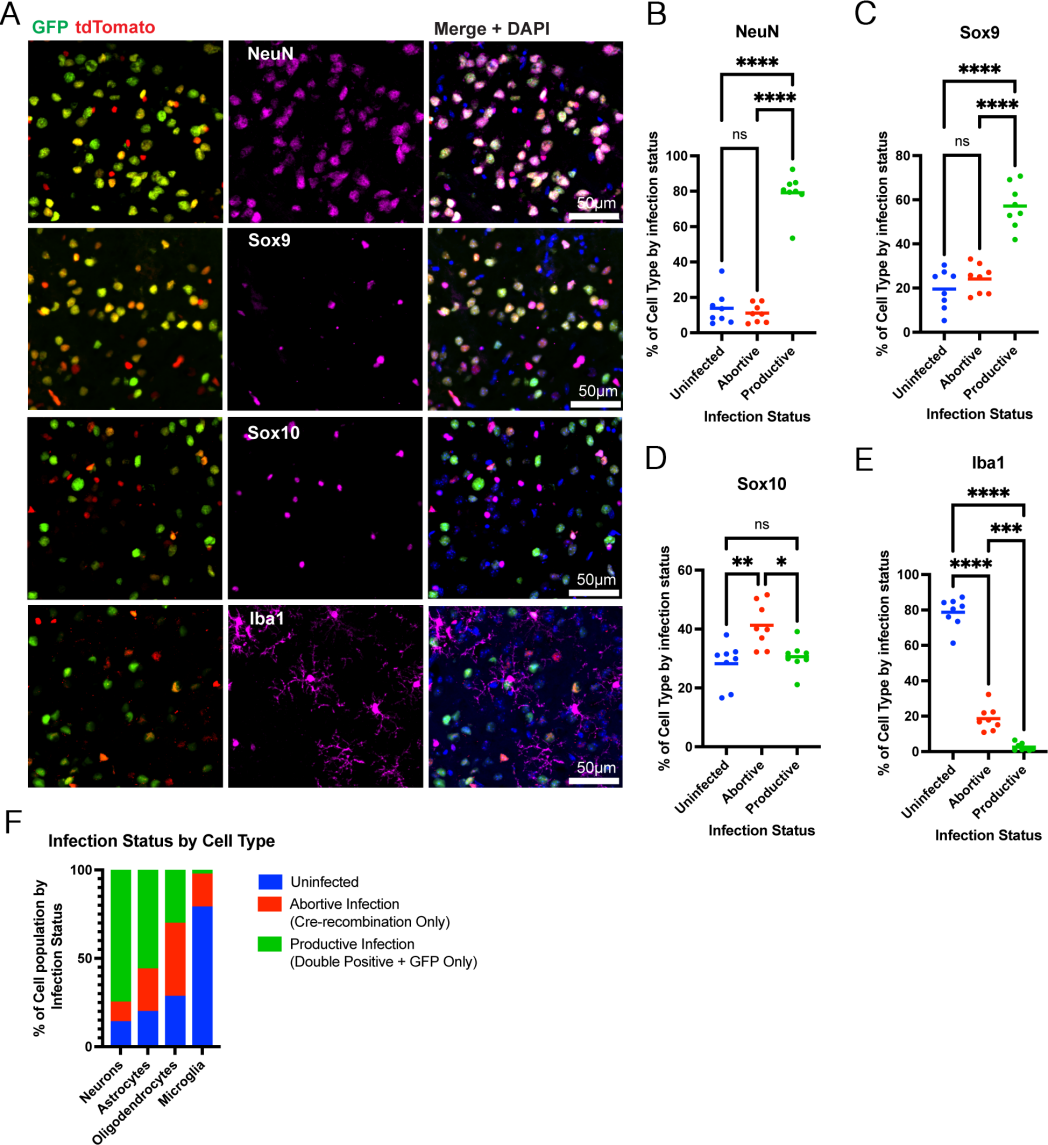
Infection status by cell type with DL-VSV. (*A*) Representative images of IHC for cell type-specific markers (magenta) depicted with infection markers, GFP and tdTomato (Cre recombination). Semiautomated quantification of images for (*B*) NeuN, (*C*) Sox9, (*D*) Sox10, (*E*) Iba1. (*F*) Summary of the proportion of each type of infection in each cell type. Infection statuses are uninfected (tdTomato-/GFP-), abortive infection (tdTomato+/GFP-), and productive infection (tdTomato+/GFP+ and tdTomato-/GFP+). The labels correspond to the following markers; neurons (NeuN), astrocytes (Sox9), oligodendrocytes (Sox10), and microglia (Iba1). Images were taken from sagittal sections 16 h postinjection of DL-VSV in the striatum. The graphs display means for each metric. Each point is from a separate animal (4 males, 4 females) with an average of 2 to 4 images per animal. One-way ANOVA was performed with Tukey’s multiple comparison test for statistical analysis. * ≤0.0332, ** ≤0.0021, *** ≤0.0002, **** ≤0.0001.

To determine the response of different cell types to viral infection, DL-VSV was used to infect the striatum in Ai75d mice, which were then assayed using cell type-specific markers. The markers used were NeuN for neurons (22), Sox9 for astrocytes (23), Iba1 for microglia (24), and Sox10 for oligodendrocyte-related cells, i.e. oligodendrocyte progenitor cells (OPCs) and oligodendrocytes (25, 26) (Fig. 3 and *SI Appendix*, Fig. S3). The majority of NeuN+ cells were productively infected, as indicated by GFP expression, followed by Sox9+ cells, to a much lesser extent (Fig. 3 *A*–*C*). In contrast, the predominant infection state of Sox10+ cells was abortive infection (tdTomato+, GFP-), while Iba1+ cells were overwhelmingly tdTomato-/GFP- (Fig. 3 *A*, *D* and *E*). The few Iba1+ cells that were infected, were abortively infected. These data indicate that NeuN+ cells are the most susceptible cell type to infection and replication, while glial cells display varying levels of permissiveness and susceptibility, with Sox10+ cells and, especially Iba1+ cells, being the most resistant to viral infection (Fig. 3*F*).

**Fig. 4.**
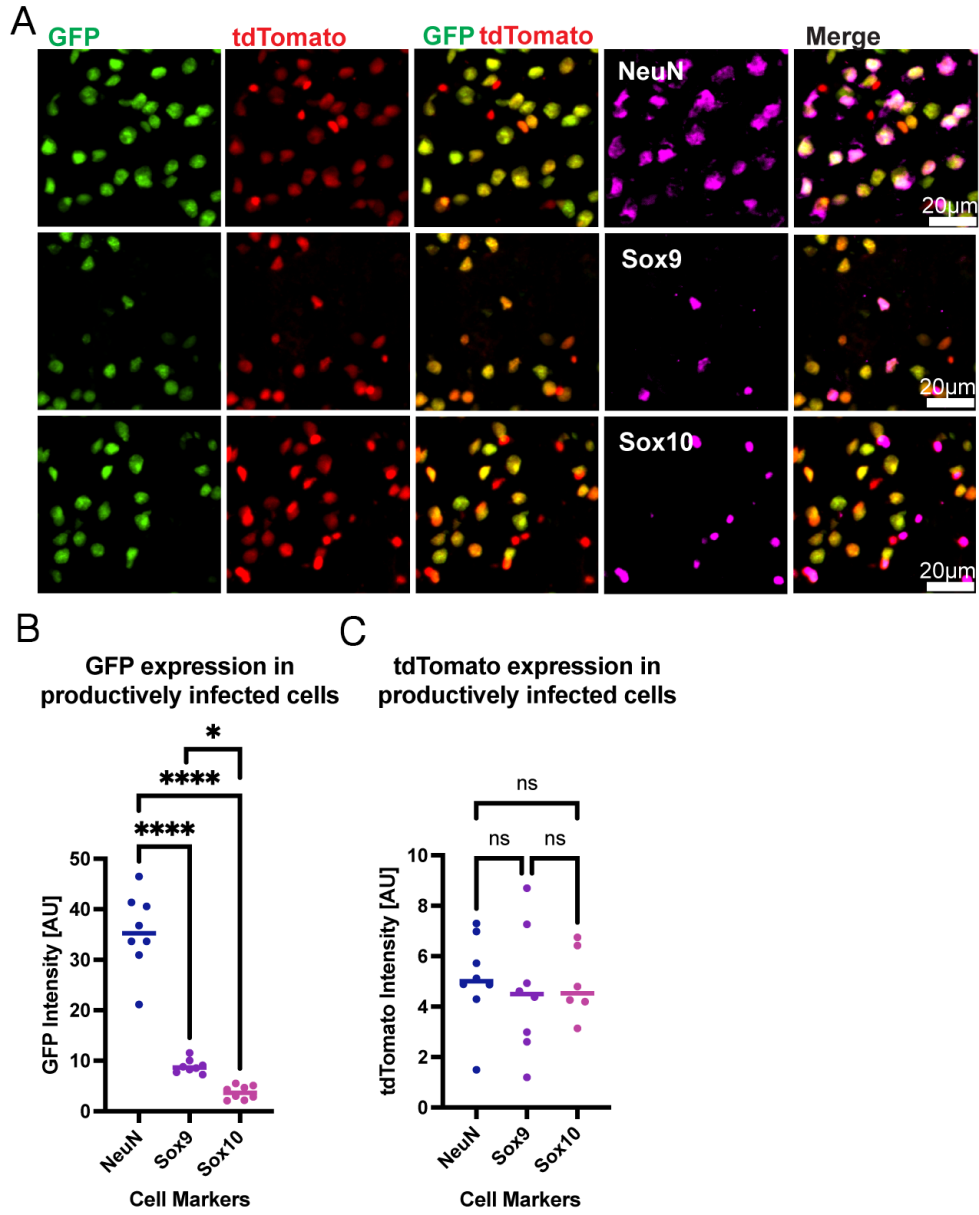
Quantification of infection markers in productively infected cells. (*A*) Magnified images of representative infected cells with infection markers [GFP and tdTomato (Cre recombination)] and IHC of cell type markers (magenta). Integrated intensity of (*B*) GFP, or (*C*) tdTomato, in productively infected cells. Integrated fluorescent intensity represented in arbitrary units generated by CellProfiler. Images taken 16 h postinfection. Graphs display means for each metric. Each point is from a separate animal (4 males, 4 females) with an average of 2 to 4 images per animal. One-way ANOVA was performed with Tukey’s multiple comparison test for statistical analysis. * ≤0.0332, ** ≤0.0021, *** ≤0.0002, **** ≤0.0001.

In addition to quantifying GFP as a binary output, it was possible to assess the level of GFP expression as an indication of the level of replication, as its mRNA and protein levels would increase with genome replication. The integrated GFP intensity in individual cells revealed that productively infected neurons support a significant amount of viral replication, as compared to either of the glial cell types (Fig. 4 *A* and *B*). Among the glial cells, Sox10+ cells expressed a lower level of GFP than Sox9+ cells. All cell types exhibited comparable expression of tdTomato (Fig. 4 *A*–*C*). Iba1+ were not assessed for GFP expression level due to the very low number of cells expressing GFP.

### Glial Cells Expressed More ISGs than Neurons.

**Fig. 5.**
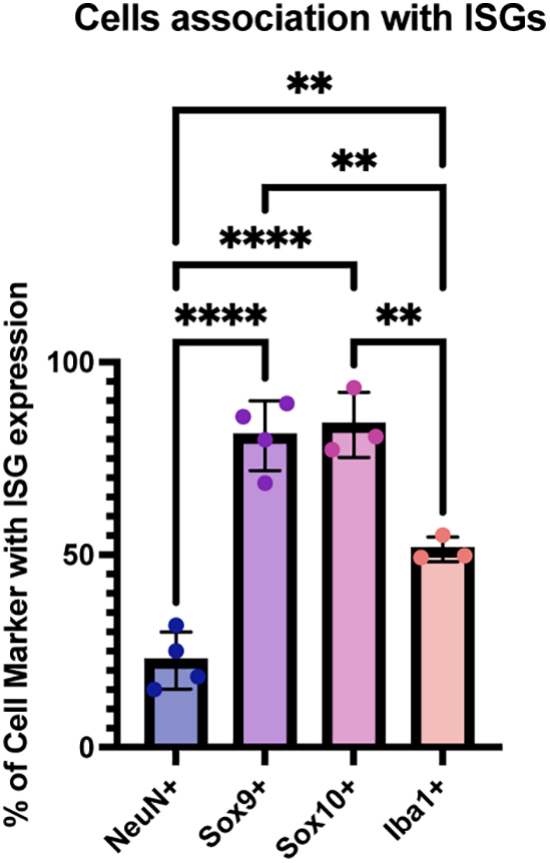
Interferon-associated gene (ISG) expression in infected regions by cell type. Pooled HCR-FISH probes detecting *ISG15, RSAD2,* and *IFIT3* were used to determine ISG expression and association with cell types in infected brains. Automated quantification of ISG expression by cell type. Images taken 16 h postinfection. Graphs display means for each metric. Each point is from a separate animal (2 males, 2 females) with an average of 2 to 4 images per animal. One-way ANOVA was performed with Tukey’s multiple comparison test for statistical analysis. * ≤0.0332, ** ≤0.0021, *** ≤0.0002, **** ≤0.0001.

One potential reason for differential infection outcomes could be the expression of ISGs. VSV is known to be sensitive to IFN and specific ISGs (12, 27–29). In order to investigate this possible correlation, HCR-FISH was conducted using a pooled probe set for 3 ISG transcripts (*RSAD2, IFIT3,* and *ISG15).* A significant colocalization of ISG transcripts with glial cells was seen, with Sox9+ and Sox10+ cells having a comparable percent of ISG positivity, and Iba1+ cells having a lower percentage. Few FISH signals were seen in NeuN+ cells (Fig. 5 and *SI Appendix*, Fig. S4*A*).

To examine whether there was a correlation of ISG expression and viral replication within productively infected cells, GFP intensity relative to ISG transcript level was analyzed. When ISG transcripts were not present in a cell, there was greater GFP signal in both NeuN+ and Sox9+ cells (*SI Appendix*, Fig. S4 *B* and *C*). The intensity of GFP did not significantly change relative to the presence or absence of ISG transcripts within Sox10+ cells (*SI Appendix*, Fig. S4*A*).

### Primary *IFNb* Induction Within Glial Cells Changes Over Time.

**Fig. 6.**
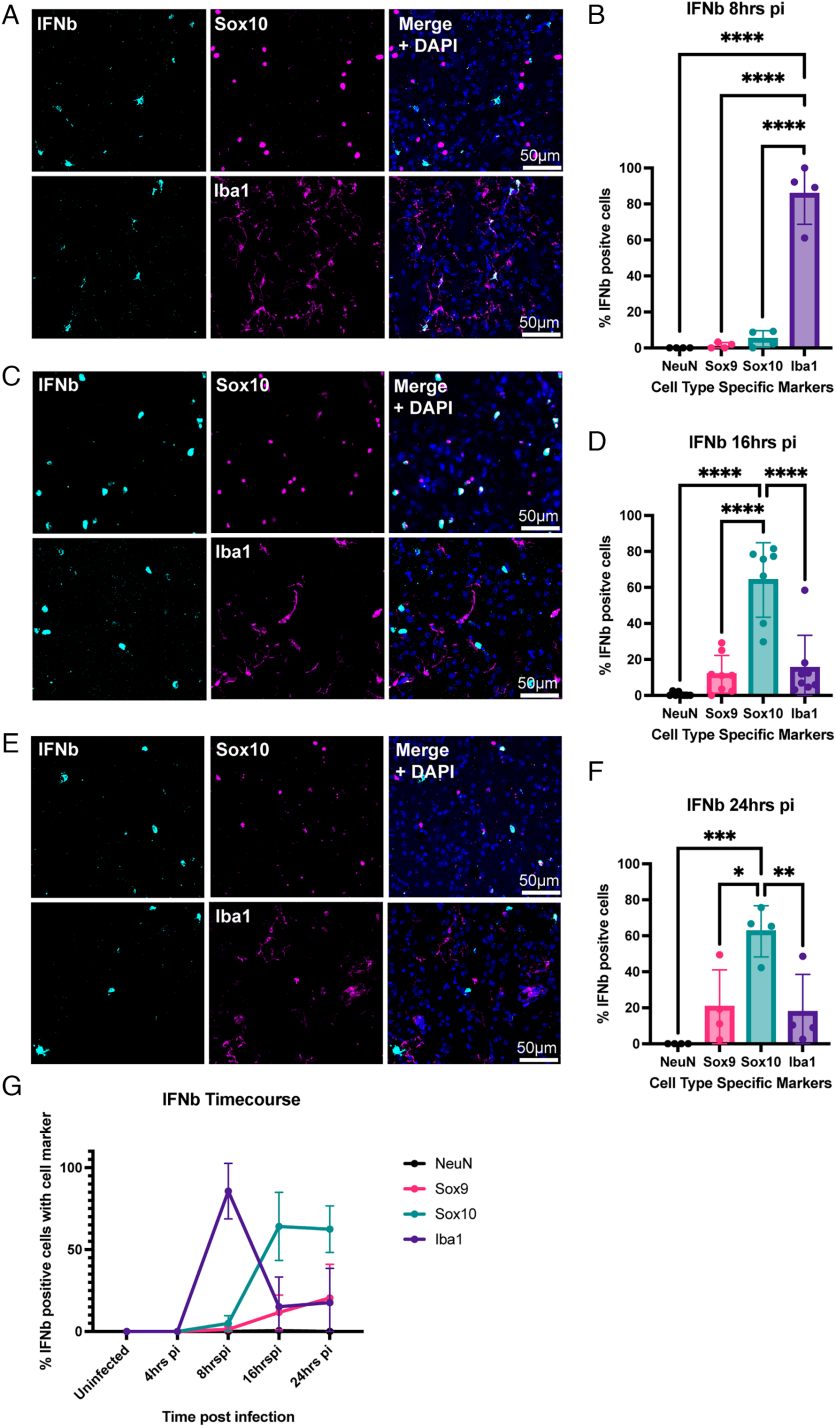
*IFNb* expression dynamics by cell type. HCR-FISH was used to detect *IFNb* over a 24 h time course. Representative IHC images and quantification for Sox10 and Iba1 markers with *IFNb* HCR-FISH at (*A*, *B*) 8 h, (*C*, *D*) 16 h, and (*E*, *F*) 24 h postinfection. (*G*) Summary of *IFNb* expression kinetics in each cell type. The graph displays means for each metric. Each point is from a separate animal with an average of 2 to 4 images per animal. One-way ANOVA was performed with Tukey’s multiple comparison test for statistical analysis. * ≤0.0332, ** ≤0.0021, *** ≤0.0002, **** ≤0.0001.

Given the sensitivity of VSV to IFN, and the observations above regarding ISGs, we used FISH to probe for the expression of IFNs. *IFNb* was seen in the infected, but not the uninfected, striatum, in both the white matter and gray matter at 16hpi (*SI Appendix*, Fig. S5 *A*–*C*). In order to determine the kinetics of its expression, a time course for IFNb expression was carried out, at 4, 8, 16, and 24 hpi. *IFNb* RNA was first detected at 8hpi and remained detectable through 24hpi (*SI Appendix*, Fig. S6 *A* and *B*). Previous studies have implicated both astrocytes and microglia in the expression of IFNb upon VSV infection in murine brain tissue (8, 12, 30). We used cell type markers and FISH for *IFNb* to determine the cell types that expressed it at each timepoint. At 8 h postinfection, Iba1+ cells were found to be the predominant cells with *IFNb* expression (Fig. 6 *A* and *B*). At 16 and 24 h postinfection, Sox10+ cells were the main cell type that expressed *IFNb* (Fig. 6 *C*–*F*). (Fig. 6*G*). NeuN+ cells did not express *IFNb*, and a minor percentage of *IFNb+* cells were Sox9+ (Fig. 6*G* and *SI Appendix*, Fig. S7).

**Fig. 7.**
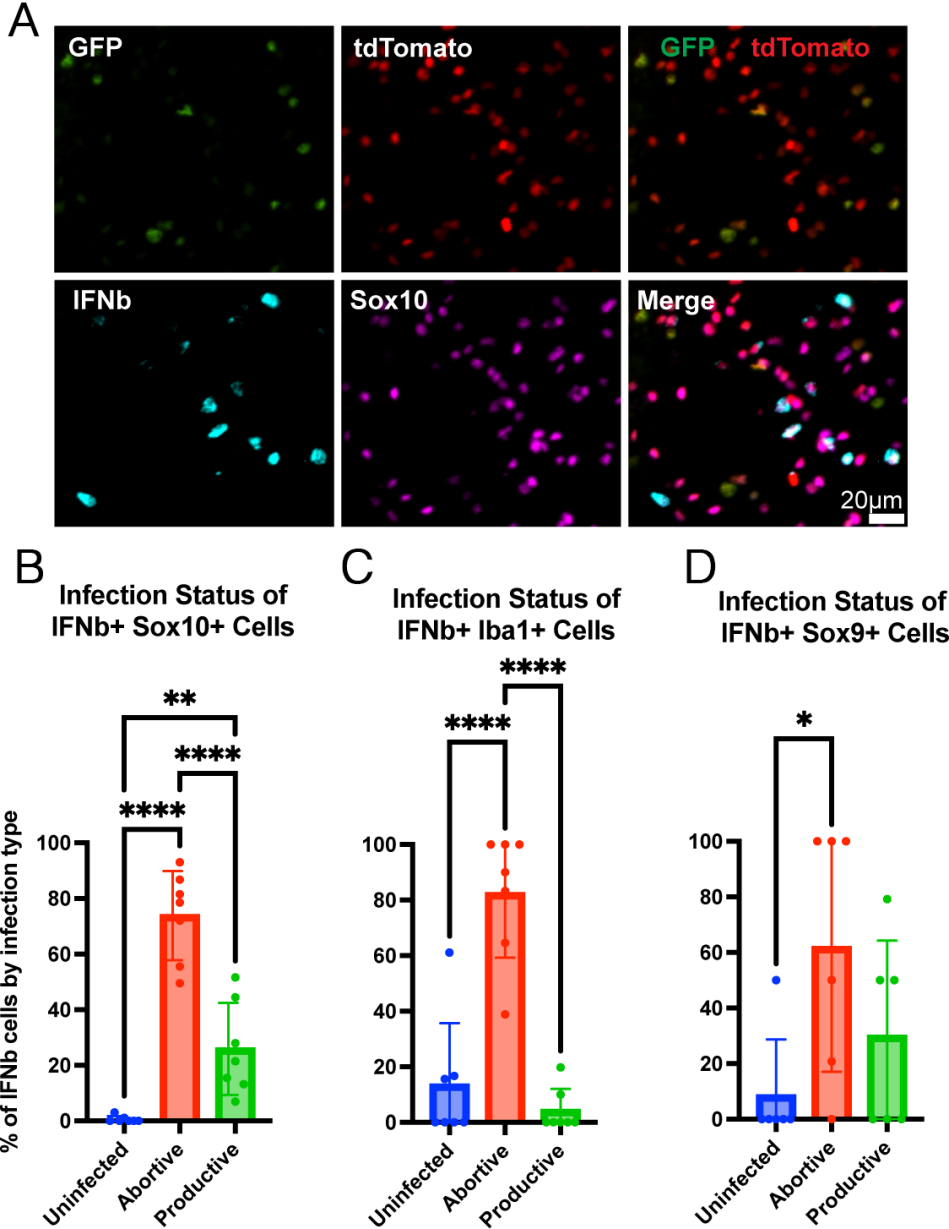
Infection status of *IFNb*-producing cells. (*A*) Representative images of infection markers, GFP and tdTomato (Cre-recombination), along with HCR -FISH signal for *IFNb* (cyan), and IHC for Sox10 (magenta) 16 h postinfection with DL-VSV. The graphs depict quantification of *IFNb*-positive cells of each cell population: (*B*) oligodendrocytes, (*C*) microglia, (*D*) astrocytes. Quantifications are taken from 16 h postinfection timepoint. Graph display means for each metric. Each point is from a separate animal with an average of 2 to 4 images per animal. One-way ANOVA was performed with Tukey’s multiple comparison test for statistical analysis. * ≤0.0332, ** ≤0.0021, *** ≤0.0002, **** ≤0.0001.

Recently, it has been suggested that abortively infected cells are one of the primary regulators of the innate immune response (8). Furthermore, it has been proposed that the innate immune system has evolved to detect infection infidelities (10). The DL-VSV allows detection of the infection status relative to *IFNb*, as a marker of the innate immune system. At 16 h postinfection, the most abundant *IFNb* expressing cells, the Sox10+ cells, were predominantly abortively infected (Fig. 7 *A* and *B*). This pattern persisted in the case of microglia and astrocytes, despite constituting less than a quarter of the *IFNb-*expressing cells (Fig. 7 *C* and *D*). A high enough level of expression of tdTomato and GFP was not present at 8hpi to assess infection status at this time point.

### IFN Receptor KO Did Not Significantly Change the Primary Infection Outcomes.

**Fig. 8.**
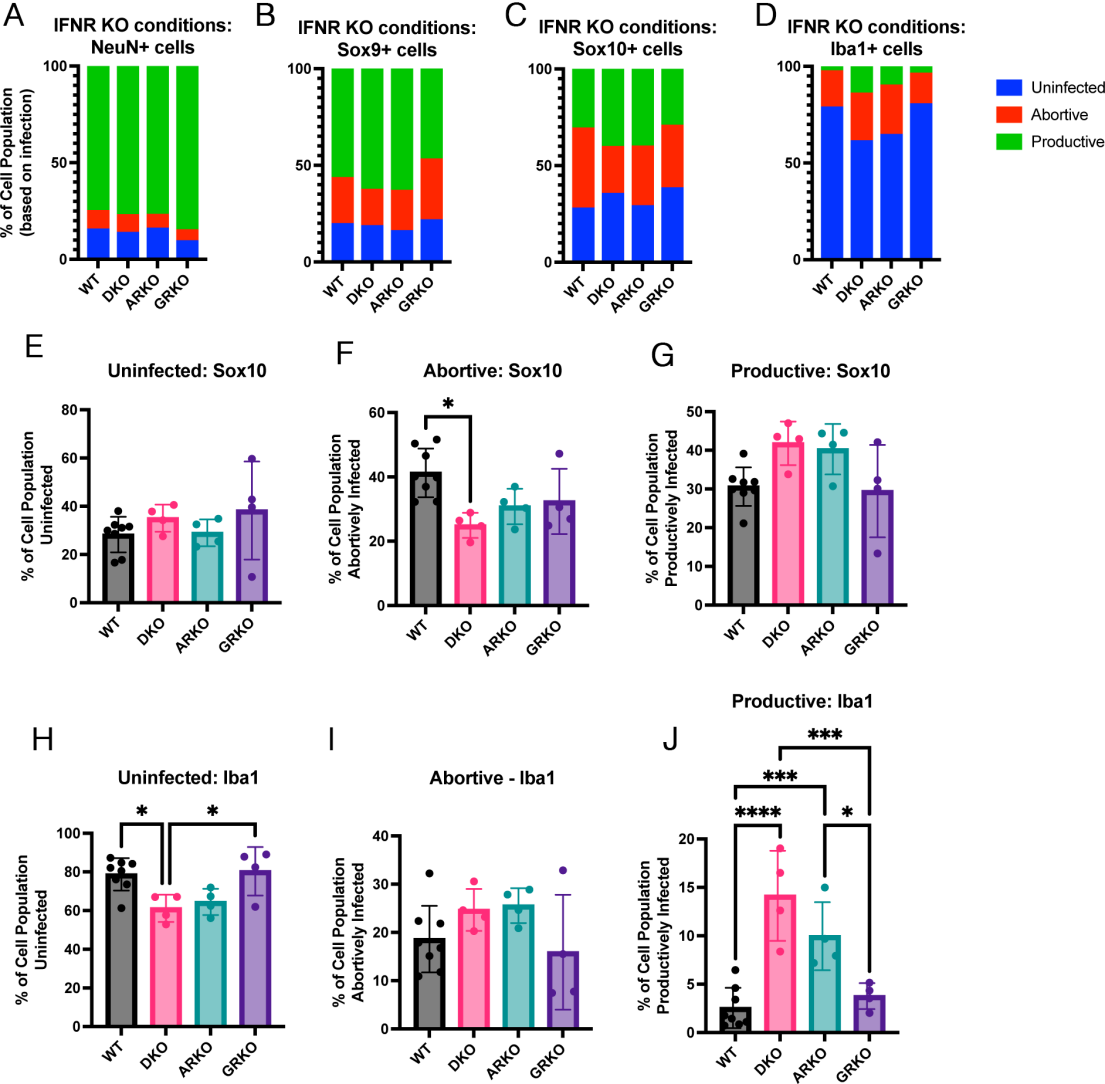
IFN receptor KO genotype and infection outcomes. IFNAR KO, IFNGR KO, or IFNDR KO mice with floxed fluorescent reporter were infected with DL-VSV and the brains were harvested 16 h postinfection. Infection status of all cell types was compared across IFN receptor KO conditions. (*A*) NeuN+ neurons, (*B*) Sox9+ astrocytes, (*C*) Sox10+ oligodendrocytes, (*D*) Iba1+ microglia. Sox10+ cells were compared across IFN receptor KO conditions within (*E*) uninfected, (*F*) abortive infection, and (*G*) productive infection. Similarly, Iba1+ cells were compared across IFN receptor KO conditions within (*H*) uninfected, (*I*) abortive infection, and (*J*) productive infection. Graphs display means for each metric. Each point is from a separate animal, 2 males and 2 females, with an average of 2 to 4 images per animal for all timepoints except 16 h postinfection (4 males and 4 females for wildtype). Nested One-way ANOVA was performed with Tukey’s multiple comparison test for statistical analysis. * ≤0.0332, ** ≤0.0021, *** ≤0.0002, **** ≤0.0001.

Given the known sensitivity of VSV to IFN, we sought to determine the contribution of IFN signaling to abortive versus productive primary infection. Mice lacking Type I and/or Type II interferon receptors (IFNAR and IFNGR KOs, respectively) should have reduced IFN responses. IFNR KO mice were crossed with the Ai75d mice to probe the outcome of DL-VSV infection in this setting (*SI Appendix*, Fig. S8*A*). To investigate the levels of IFN signaling in such IFNR KO’s, we examined the expression of the same set of ISG transcripts as used in Fig. 5. A substantial reduction of ISG expression was seen in IFNAR−/− mice and the IFNAR−/−/IFNGR−/− (dKO mice (*SI Appendix*, Fig. S8*B*). There was no noticeable alteration in ISG expression in the IFNGR−/− mice. The infection outcome in various IFN receptor KO’s did not change in NeuN+ or Sox9+ cells (Fig. 8 *A* and *B*). However, in the dKO, abortively infected Sox10+ cells decreased slightly, without a significant increase in productively infected cells (Fig. 8 *F* and *G*). Plotting the ratio of abortive infection to total infection revealed a slight shift in the dKO compared to wildtype and IFNGR KO (*SI Appendix*, Fig. S9*D*). In Iba1+ cells, there was a more pronounced shift of primary infection outcomes. Specifically, there was a significant decrease in uninfected Iba1+ cells with an increase in productive infection in the dKO and IFNAR KO conditions as compared to wildtype and IFNGR KO conditions (Fig. 8 *H* and *J*). This shift also was evident in the infection ratios of Iba1+ cells (*SI Appendix*, Fig. S9*E*). However, although there were slight changes in infection outcomes in certain glial cell populations, the predominant pattern of infection of each cell type persisted across the different KO’s (Fig. 8 *A*−*D*).

Even if IFN signaling did not have a major impact on infection outcome, we investigated whether it influenced replication by quantifying GFP intensity in productively infected cells. In Sox9+ and Sox10+ cells there was a modest yet statistically significant increase in GFP expression intensity in the dKO when compared to wildtype (*SI Appendix*, Fig. S9 *B* and *C*). However, no difference was observed in neurons (*SI Appendix*, Fig. S9*A*), in agreement with the previous findings that indicated limited expression of ISGs in neurons compared to glial cells (Fig. 5). The assessment of microglia was not possible due to the low number of productively infected microglia in the wildtype condition.

## Discussion

The data presented here describe a system that allowed us to track virus–host infection dynamics in vivo. We categorized various cell types by their infection states and identified specific subsets of cells that exhibited abortive infection. We were able to recognize a temporal progression of the cell types with induced IFN expression, demonstrating an underappreciated expression pattern of IFN by Sox10+ cells.

### Use of Viral and Host Readouts of Infection to Track Virus–Host Dynamics.

One of the most fundamental questions about a virus is its tropism. Common approaches to address this question include monitoring the abundance of viral proteins or genetic material. The presence or absence of these viral products can indicate whether a cell is susceptible to viral infection and begin to define viral tropism. However, depending on the sensitivity of the detection, defining viral tropism from a viral readout might not capture instances when a virus infects a cell but is unable to undergo significant replication. Thus, a viral readout itself is insufficient in identify intermediate/abortive states of infection.

In an effort to better capture infection dynamics, we incorporated the Cre-lox system to enable a host readout of infection. Previous viral approaches have utilized Cre-recombination for the permanent labeling of infected cells in the cases of fusion assays, viral tracing, and even for capturing abortive infection events (8, 31–34). For convenience, we paired the Cre recombinase readout with a typical fluorescent readout of viral infection, H2B-eGFP. This combination of infection readouts builds upon previous systems by combining a viral and host readout of infection in a single virus, thus standardizing the output for easier comparison, while also mitigating any technical limitations by separately assessing such readouts. Moreover, the use of nuclear-localized signals for both infection readouts facilitates segmentation, colocalization of markers, and automated quantification.

We initially posited that due to the ubiquitous expression of LDLR, the receptor for VSV-G, every cell should be susceptible to infection (35). Previous investigations conducted in our lab and others have demonstrated that VSV is capable of productively infecting neurons (36–38). In relation to other cell types of the CNS, VSV’s tropism is more variable. While there have been reports of wildtype VSV infecting astrocytes and microglia (39), we observed a more complex phenotype when examining primary infection with the DL-VSV. In general, we observed that glial cells are more resistant to DL-VSV infection compared to neurons. Astrocytes were primarily productively infected, Sox10+ cells had a higher proportion of abortive infection, and microglia were mostly uninfected, with some exhibiting abortive infection. Some of the differences between our results and previous studies may be attributed to the fact that we assessed primary infection and utilized an attenuated VSV lacking VSV-G and containing a mutated M (19, 40, 41). While our specific observations regarding the infection outcome of each cell type might be unique to our virus and infection parameters, other viral systems can benefit from adopting the dual labeling strategy to uncover intermediate states of infection to better define viral tropism.

### Expression of IFN by Different Glial Cell Types.

It is widely recognized that VSV has the ability to stimulate an innate and adaptive immune response upon infection in the CNS (42). Recently, it is becoming apparent that the coordination of this immune response necessitates interactions among multiple cell types (32, 43–46). In the case of VSV, both microglia and astrocytes have been implicated as the primary producers of IFN in the early innate immune response in the CNS (8, 12, 30). The extent to which each glial cell type contributes to this IFN induction depends upon various factors, such as the viral strain and the specific site of inoculation. Our objective was to determine the relative contribution of different cell types in the CNS to IFN induction by DL-VSV over time.

To our surprise, not only did we observe *IFNb* production in Sox10+ cells, but we also noticed that there was a switch from microglia to Sox10+ cells in the induction of *IFNb*. Apart from one study conducted in vitro, Sox10+ cells have not been linked with Type I IFN induction during viral infection (47). This may be due to the relatively lower basal levels of pathogenic recognition receptors (PRRs) in these cells compared to other glial cells (48). The fact that we observed Sox10+ cells producing *IFNb* could be a result of the examination of different timepoints and the use of the attenuated DL-VSV. Using an attenuated virus may enable Sox10+ cells, which might be overrun by a more virulent virus, to detect and induce an IFN response. Moreover, it is common to assay only one time point following infection, which would not allow an appreciation of the response of different cell types as an infection proceeds. Previous studies have reported that, at different time points, microglia and astrocytes produce specific Type I IFN species after mouse hepatitis virus (MHV) infection in the brain (45). Additionally, upon poly I:C administration in the brain, microglia exhibit an acute induction of *IFNb,* while oligodendrocytes begin to express more PRRs later (48). The transition of microglia to Sox10+ cells as the primary producers of *IFNb* may be due to a delay in the accumulation of PRRs by Sox10+ cells. Once they accumulate a threshold level or repertoire of PRRs, they may be able to respond to viral infection themselves. An interesting possibility that remains to be tested is whether microglia are necessary for the induction of *IFNb* in Sox10+ cells.

Regardless of the specific cell type responsible for inducing IFN, our findings indicate that the majority of *IFNb* production originates from abortively (tdTomato+ only) infected cells. This is a crucial observation because, without the Cre-recombination readout, we might have concluded that IFNb was expressed from “uninfected” cells. Furthermore, these data show a similar response to VSV as to RABV, wherein abortively infected astrocytes were shown to produce IFNb (8). Additionally, these findings align with the more recent idea that the innate immune system has evolved to detect infection infidelities (10). Here, the cells which could not effectively replicate the virus produced the most cytokines.

One potential consideration that must be taken into account is the possibility that Cre-recombination triggers an IFN response, as a previous study showed that Cre-recombination can activate the cGAS–STING innate immune response (49). STING is an upstream regulator of inflammation and can induce the expression of Type I IFNs (50). However, if the induction of IFN production via STING were indeed caused by Cre-recombination, we would have expected that *IFNb* induction would occur in more cells, regardless of cell type or type of infection. We did not observe such a response.

### Factors Contributing to Primary Infection Outcomes.

VSV is known to be sensitive to the IFN-induced antiviral state. We and others have demonstrated that the absence of IFNAR enhances VSV pathogenesis and spread in the CNS (12, 27, 44, 45). Previous reports have shown that Type I IFN signaling contributes to the tropism of measles virus and tick-borne flaviviruses in CNS cells (51, 52). In these cases, IFNAR−/− led to an increase in the susceptibility of microglia to infection. Additionally, variations in RABV strains, which have differences in the control of antiviral responses, have been observed to influence tropism (4, 5). While these previous studies used viruses that were replication-competent and able to spread, we wondered whether primary infection of VSV also could be influenced by IFN signaling.

DL-VSV infections showed that cells with a higher level of ISGs were more resistant to viral infection. However, when we disrupted IFN signaling, we observed only a slight shift toward more productive infection of Sox10+ cells and microglia. The predominant infection state of each cell type did not change in the presence or absence of Type I or Type II IFN signaling. One potential explanation for the absence of a change in tropism is that we were specifically monitoring primary infection with DL-VSV. Here, the earliest detection of *IFNb* occurred at 8hpi. Consequently, IFN-induced responses would only occur between approximately 8hpi and the time of tissue harvest at 16hpi. We postulate that the IFN signaling is occurring too late to influence primary infection. If we were to employ a replication-competent DL-VSV capable of spread, IFN signaling could potentially impact secondary or tertiary infections, thereby leading to differences in abortive versus productive infections.

Since IFN signaling is likely occurring too late to influence primary infection outcomes, other factors must be contributing. A recent study conducted with human cytomegalovirus (HCMV) identified an association between primary infection outcome and the expression of ISGs. Specifically, cell-intrinsic ISG expression was the main factor dictating primary infection outcome rather than an induced response (53). In our study, even in IFNAR−/− mice, we still observed low levels of ISG transcripts that could potentially contribute to an antiviral effect. These data indicate that IFN signaling does not significantly influence the outcomes of primary infection; instead, other intrinsic factors are likely responsible for these differential infection outcomes (54).

### Future Directions.

In this study, the distinction between OPCs and mature oligodendrocytes was not made while using the Sox10 marker. Consequently, it is worth determining whether the abortive infection phenotype and *IFNb* induction are more closely associated with OPCs or mature oligodendrocytes. Furthermore, it is possible that some of our observations might be unique to the modifications we implemented with the DL-VSV. Therefore, the adoption of a dual-labeling strategy with different VSV mutants and other viruses on a broader scale will aid in elucidating early virus–host infection dynamics.

## Methods

### Virus Cloning and Production.

The DL-VSV (rVSV-Cre-N-P-Md-H2B-eGFP-L) was generated from a VSVΔG backbone (55). The Cre, H2B-eGFP, and Md [double mutation Matrix, M33A and M51R (19)] were introduced into the backbone by Gibson assembly of gBlock fragments with a restriction digested linearized vector. Viruses were rescued as previously described (20, 55). The DL-VSV was expanded by first transfecting 293Ts in a 10 cm dish at 70% with pCAG-VSV-G. 36 h posttransfection, a fresh plate of 293 T cells were infected at an MOI of 0.01 with the rescued DL-VSV. Viral supernatants were harvested 48 h postinfection and spun down to pellet cellular debris at 4,000 rpm for 15 min at 4C. Supernatant was collected and concentrated using a SW28 rotor at 20K rpm for 2 h at 4 °C. Supernatant was discarded, and the viral pellet was resuspended in DMEM overnight at 4 °C. The resuspended virus was then overlaid on a 10% sucrose cushion (10 ml) for centrifugation in a SW40 rotor for 2 h at 4 °C. Supernatant was discarded, and the viral pellet was resuspended in 1xPBS and subsequently stored at −80 °C.

### Primary Transcription In Vitro Experiments.

The virus lacking VSV nucleocapsid (N), phosphoprotein (P), and glycoprotein (G); (rVSV-Cre-dN-dP-M-eGFP-L) was generously donated by Dr. Xiang Ma (Cepko lab). 293Ts in a 6-well plate were transfected at 70% confluency using PEI with plasmids expressing N, P, L, and Cre reporter; N, P, and L; or only Cre reporter plasmid. 36 h posttransfection, the cells were infected at an MOI of 3 with the VSV lacking N and P. Images were taken 24 hpi showing the GFP and tdTomato readouts (Cre-recombination readout) separately.

### In Vitro Cre Transfer Experiment.

The experimental setup is depicted in *SI Appendix*, Fig. S2*A*. 293Ts in a chamber slide were all transfected using PEI with a Cre reporter plasmid (from Dr. Yashodhan Chinchore, Cepko Lab). Concurrently, two sets of other 293Ts were infected at an MOI of 3. 36 h posttransfection of the chamber slide wells, one chamber was infected with DL-VSV, another chamber was left uninfected, the 3rd chamber was not infected but rather infected cells from the other 293Ts were detached with Trypsin-EDTA and overlaid on top of the transfected 293Ts, the 4th condition also was not infected and instead previously infected 293Ts were lysed for 20 min (lysis buffer: 150 mM NaCl, 1% Triton X-100, 50 mM Tris ph 8.0), spun down, and the supernatant was added to transfected 293Ts in the chamber well. Images are depicted with separated channels showing tdTomato and GFP followed by a merge with DAPI.

### Mouse Strains.

Twelve-week-old Ai75d mice (025106, The Jackson Laboratory) which contain a floxed tdTomato nuclear reporter were used for all time course experiments and wildtype comparisons in the IFN receptor KO experiments. The IFNb time course experiment was composed of 2 males and 2 females for each timepoint (uninfected, 4 hpi, 8 hpi, 16 hpi, 24 hpi). For the IFN receptor KO experiments, the Ai75d mice were crossed with IFNAR−/−/ (028288, The Jackson Laboratory), IFNGR−/− (003288, The Jackson Laboratory), or IFNAR−/−/IFNGR−/− (029098, The Jackson Laboratory) to create a mouse line that contained a floxed fluorescent reporter and lacked the IFNa/b receptor, IFNg receptor, or both, respectively. Mice used for the KO experiments were 12-16 wk old and consisted of 2 males and 2 females per each condition. Mice were bred under BSL-1 conditions within the Harvard Center for Comparative Medicine facilities.

### Stereotaxic Injections.

All brain injections were performed under BSL-2 conditions in the Harvard Center for Comparative Medicine facilities. The animals were injected using pulled capillary microdispensers (Drummond Scientific) using the following coordinates for the striatum; A/P 1.0 from bregma, L/M 1.8, D/V −2.5. We injected 200 nl of DL-VSV at a concentration of 1.3 × 10^9 PFU/ml (titered on 293Ts).

### Tissue Processing.

At the indicated times postinfection, the mice were perfused, and the brains were harvested and fixed in 4% paraformaldehyde (w/v in 1 × PBS) overnight at 4 °C. The brains were then left in a 30% sucrose solution (w/vv 1 × PBS) complemented with 1× RNase inhibitor (RNase OUT, Thermo Scientific) until the tissue was saturated and lost buoyancy (24 to 36 hpi). The brain tissue was flash frozen in O.C.T (4583; VWR) using dry ice. Frozen tissue was stored at −80 °C until sectioned. Tissue sections were made at 20 μm using a Leica CM (3050S cryostat). Only the infected regions were sectioned and mounted on PDL-coated (0.3 mg/ml) Superfrost Plus microscope slides (22-037-446; Fisher Scientific).

### VSV Genome and Transcript Detection.

Original experiments staining for VSV were performed using SABER-FISH while later experiments used HCR-FISH (Fig. 2). Images of VSV genomes and transcripts in white matter vs gray matter regions were from the SABER-FISH protocol. Viral genome detection in single cells was performed using HCR-FISH. All incubations were performed on the microscopy slides with a hybridization coverslip (HybriSlip, Grace Bio-Labs) in a humidified chamber (23-769-522; Fisher Scientific). Any incubations at elevated temperatures were conducted within a hybridization oven.

#### *SABER-FISH*.

SABER-FISH was performed as described previously (56). Briefly, frozen tissue sections were rehydrated with 3 × 5 min in 1X PBSTw (PBS + 0.1% Tween 20), washed with a wash-hybridization solution (40% formamide + 1% Tween 20 + 2X saline sodium citrate [SSC]) at 43 °C for 15 min, followed by a 16 h incubation at 43 °C in a hybridization solution (40% formamide +10% dextran sulfate + 1% Tween 20 + 2X SSC) containing 8.33ug/ml of FISH probes. Afterward, samples underwent 2 × 30 min washes with the wash-hybridization solution at 43 °C followed by 2 × 5 min washes in 1X SSCT at room temperature. Hybridization probes were then detected by incubating 0.2 μM fluorescent oligonucleotides (from IDT) in 1X PBS + 0.2% Tween 20 for 30 min at 37 °C. Samples were subsequently washed 2 × 5 min with 1X PBSTw + DAPI at room temperature before being mounted in Fluoromount-G mounted medium (00-4958-02; Thermo Scientific) with a glass coverslip for imaging.

#### *HCR-FISH*.

All incubations were performed on the microscopy slide with a hybridization coverslip (HybriSlip, Grace Bio-Labs) in a humidified chamber (23-769-522; Fisher Scientific). Any incubations at elevated temperatures were conducted within a hybridization oven.

All probes and reagents were designed and ordered from Molecular Instruments. HCR-FISH was performed as described previously (57). Briefly, slides were rehydrated with 2 × 5 min 1X PBST washes. Afterward, 200 ul of probe hybridization buffer was incubated with the tissue section on the slide for 10 min at 37 °C. Each slide sample was then incubated in 100 ul of hybridization solution containing 0.4 pmol of each probe set, and incubated overnight at 37°C in a humidified chamber within a hybridization oven. After the overnight incubation, the tissue section was washed in a series of solutions for 15 min each (75% probe wash buffer/25% 5X SSCT, 50%/50%, 25%/75%, 0%/100%). Amplification was conducted by applying 200 ul of amplification buffer to a section in a humidified chamber for 30 min at room temperature. Snap-cooled hairpins (6 pmol of hairpin h1 and 6 pmol of hairpin h2, heated separately at 95 °C for 90 s and cooled in a dark drawer for 30 min) were mixed with 100 ul amplification buffer and applied to tissue sections. The samples were incubated overnight at room temperature. The following day, 2 × 30 min washes of 5X SSCT were conducted at room temperature before a 10 min incubation in 1X SSCT + DAPI followed by mounting in Fluoromount-G mounting medium with a coverslip.

For multiplexed imaging with more than one FISH signal, the protocol continued with a 1X PBST wash to remove mounting medium followed by 2 × 5 min 5X SSCT washes at room temperature. For removing *IFNb* signal, dissociation strands (Molecular Instruments) were used to displace the amplifiers. A 10× concentration of dissociation strands matching the amplifier (60 pmol) were incubated in 5X SSCT for 3 to 4 h at room temperature. Samples were washed 5 × 5 min in 5X SSCT. Verification to ensure that the signal was gone was done by examination under the microscope.

For removing ISGs (or another high signal), DNAse treatment was required. After washes, samples were incubated in a 1:50 DNAse solution (18047019, Invitrogen) at 37 °C for 1 h. Initial DNAse treatment was followed with another 1:50 DNAse incubation overnight at 37 °C. A wash with 65% formamide in 2X SSC was carried out for 1 h at room temperature. The formamide solution was washed away with 2 × 5 min washes with 5X SSCT.

### Immunofluorescence.

All immunofluorescence was carried out after DNAse treatment of FISH experiments to ensure that no signal carryover occurred. Incubations were conducted in humidified slide boxes and included the use of hybridization coverslips. Primary antibody incubation occurred at 4 °C, overnight with a 1:200 dilution of antibody in blocking/staining buffer (0.1% Triton X-100, 1% BSA, 3% Donkey Serum, in 1X PBS). Washes with 1X PBS occurred at an interval of 3 × 5 min. Secondary antibody was diluted to 1:500 in the same blocking/staining solution as mentioned above for 2 h at room temperature. Samples were washed 3 × 5 min with 1X PBS before being mounted in Fluoromount-G with a coverslip.

The antibodies used were as followed: anti-NeuN (ABN91, Millipore), anti-Sox9 (AB5535, Millipore), anti-Sox10 (ab180862, Abcam), Iba1 (GTX100042, GeneTex), Donkey anti-Rabbit Alexa 647 (711-605-152, Jackson ImmunoResearch), and Donkey anti-Chicken Alexa 647 (703-605-155, Jackson ImmunoResearch).

Sections were initially screened using a Nikon Eclipse E100 upright epifluorescence microscope to identify samples with the highest level of infection. These slides were subsequently used for staining, imaging, and quantification. All imaging took place on a Nikon Ti2 inverted spinning disc microscope using the 20× objective unless stated otherwise. All images were taken using consistent laser power and excitation times within each channel.

### Image Processing and Semiautomated Quantification with CellProfiler.

#### *FIJI*.

All images were preprocessed using ImageJ/FIJI macros to streamline analysis. The macros briefly consisted of several steps: 1) running a max intensity Z projection to compress Z stack for each image set, 2) split channel, 3) running the plugin “MultistackReg” for registering serial images when necessary (GFP channel was common among each imaging session and used as reference), “Rigid body” transformation setting was used unless poor registration in which case others transformations were tested, 4) performed background subtraction with a rolling ball radius of 50.0 pixels, 5) performed autoenhanced brightness and contrast at a saturation level of 0.35 (images used for fluorescent intensity comparison used same brightness/contrast parameters for each image), 6) channels merged when more than 4 channels were present and pseudocolors were used for LUTs for color differentiation.

#### *CellProfiler*.

The infected areas of the preprocessed images (mentioned above) were outlined for quantification and subsequently separated by channels and imported into the CellProfiler pipeline for analysis. The pipeline consists of 1) performing “IdentifyPrimaryObjects” for each channel. A set of thresholds based on size and intensity were used as a baseline for each fluorescent marker/stain but were manually checked. If threshold was not sufficient, the threshold was changed and noted. 2) identified infection marker objects were related and filtered based on colocalization to obtain distinct classifications (i.e. abortive infection [tdTomato-only], double positive [tdTomato + GFP], and GFP-only]). 3) New infection marker objects were masked on cell markers and binned based on percentage overlap (e.g. 80% infection marker overlap with cell marker counted as infected cell). 4) Overlays of infection markers and cell markers were made to ensure limited errors in automated analysis. 5) Object intensity measurements for identified objects based on cell markers, genes of interests, and infection markers. 6) Cell counts and intensities were exported to a CSV file and transferred to Excel for subsequent calculations (58, 59).

## Supplementary Material

Appendix 01 (PDF)

## Data Availability

All experimental images, analysis pipelines, and plasmid information are available on Figshare (60). All other data are included in the article and/or *SI Appendix*.
