# Supplementary material for: Abortive and productive infection of CNS cell types following in vivo delivery of VSV
Source: Proc Natl Acad Sci U S A. 2024 Aug 19;121(35):e2406421121. doi: 10.1073/pnas.2406421121 (PMC11363278; doi:10.1073/pnas.2406421121)
Supplement: Supplementary file 1 — Appendix 01 (PDF) [file pnas.2406421121.sapp.pdf]

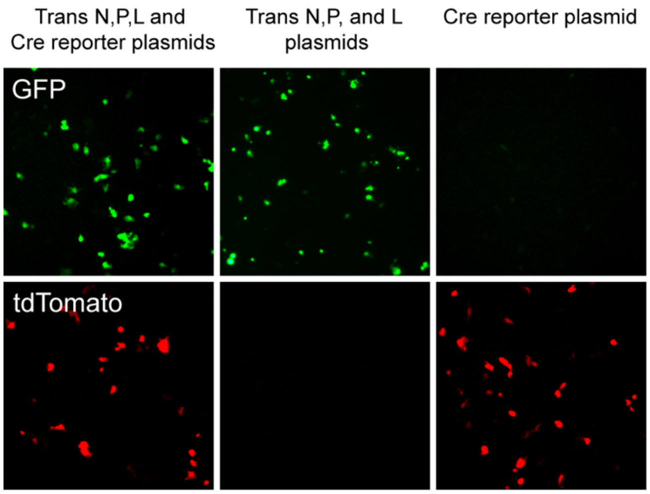

Supplemental Figure 1: Cre-recombination in the absence of viral replication

A) Modified dual labeling VSV (DL-VSV) lacking N, P, and G but expressing GFP and Cre recombinase was used to infect transfected cells. In the first set of vertical panels, plasmids encoding VSV N, P, L, and a Cre reporter were transfected into 293T cells. In the middle set of vertical panels, plasmids encoding VSV N, P, and L were transfected. In the last set, only the Cre reporter plasmid was transfected. All three sets of transfected cells were infected for 24hrs with the (DL-VSV) . Images from each condition are split, showing GFP and tdTomato (Cre recombination readout) independently.

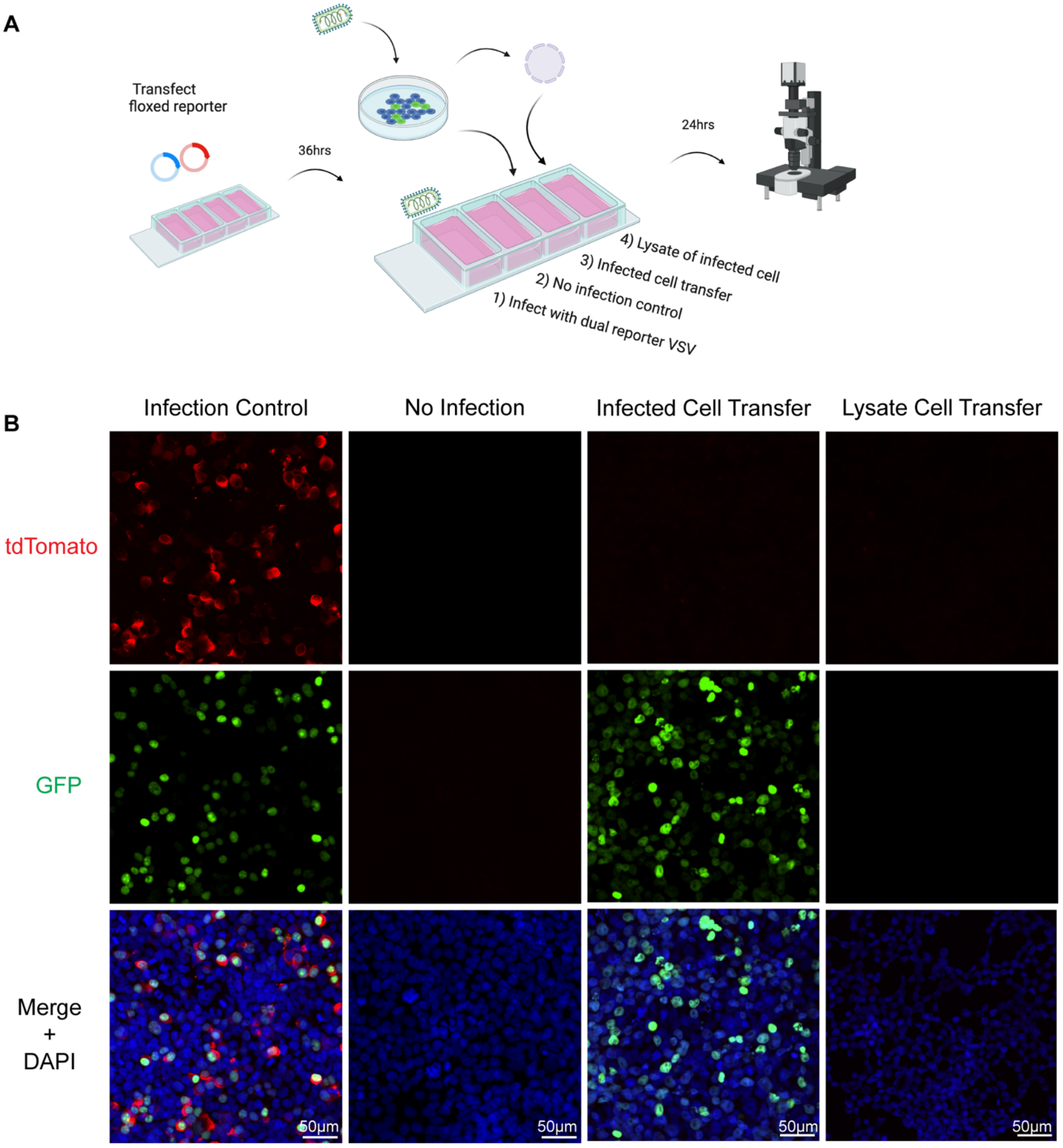

Supplemental Figure 2: Testing of Cre recombination in bystander cells *in vitro*

A) Diagram of an *in vitro* experiment testing whether Cre recombination can be induced in cells by transferred Cre activity. In all four conditions, 293T cells were transfected with a floxed tdTomato reporter plasmid. 36hrs post transfection, cells were either infected with DL-VSV, not infected, overlayed with cells previously infected (but not transfected with recombination

reporter), or overlayed with cell lysate from previously infected cells. B) Panels display GFP, tdTomato (floxed reporter), and DAPI images with a merged image of all the fluorescent channels of each condition.

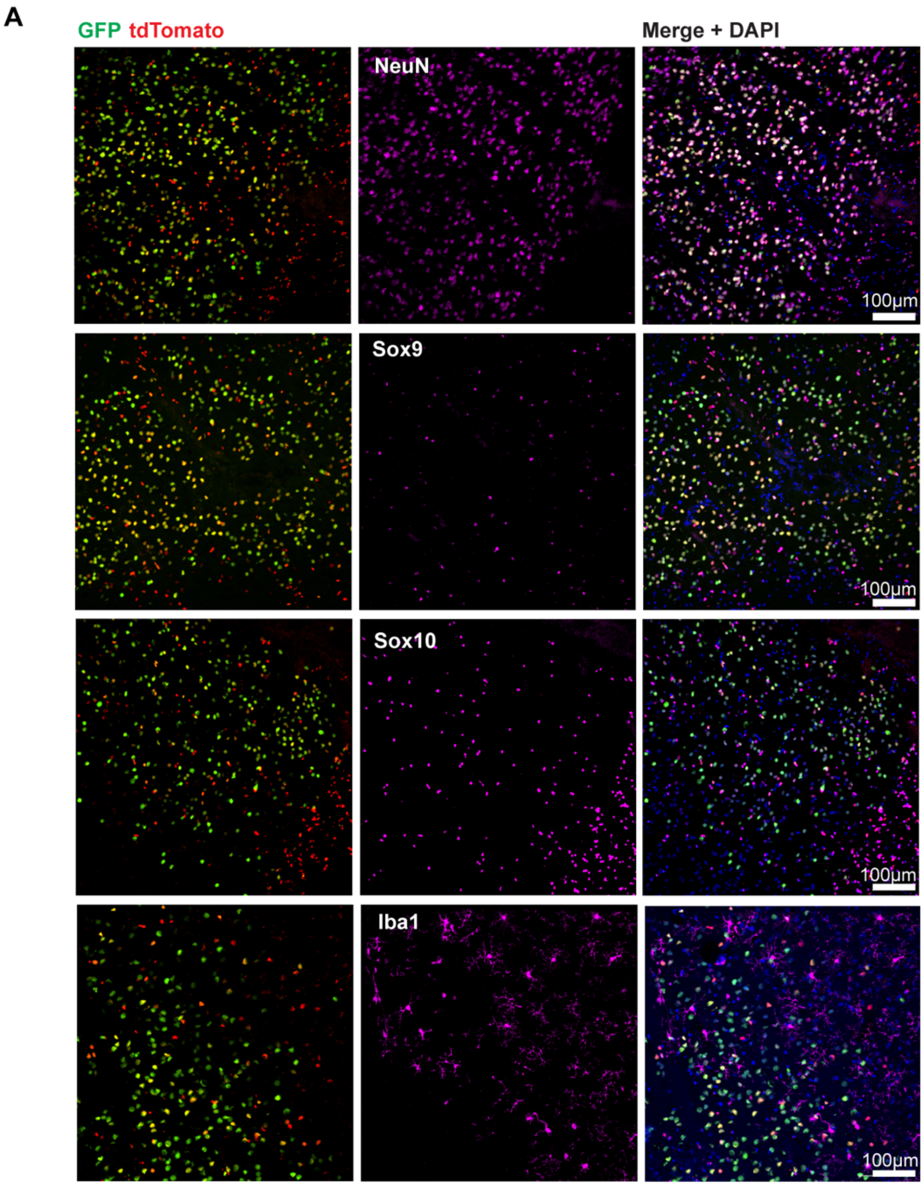

B

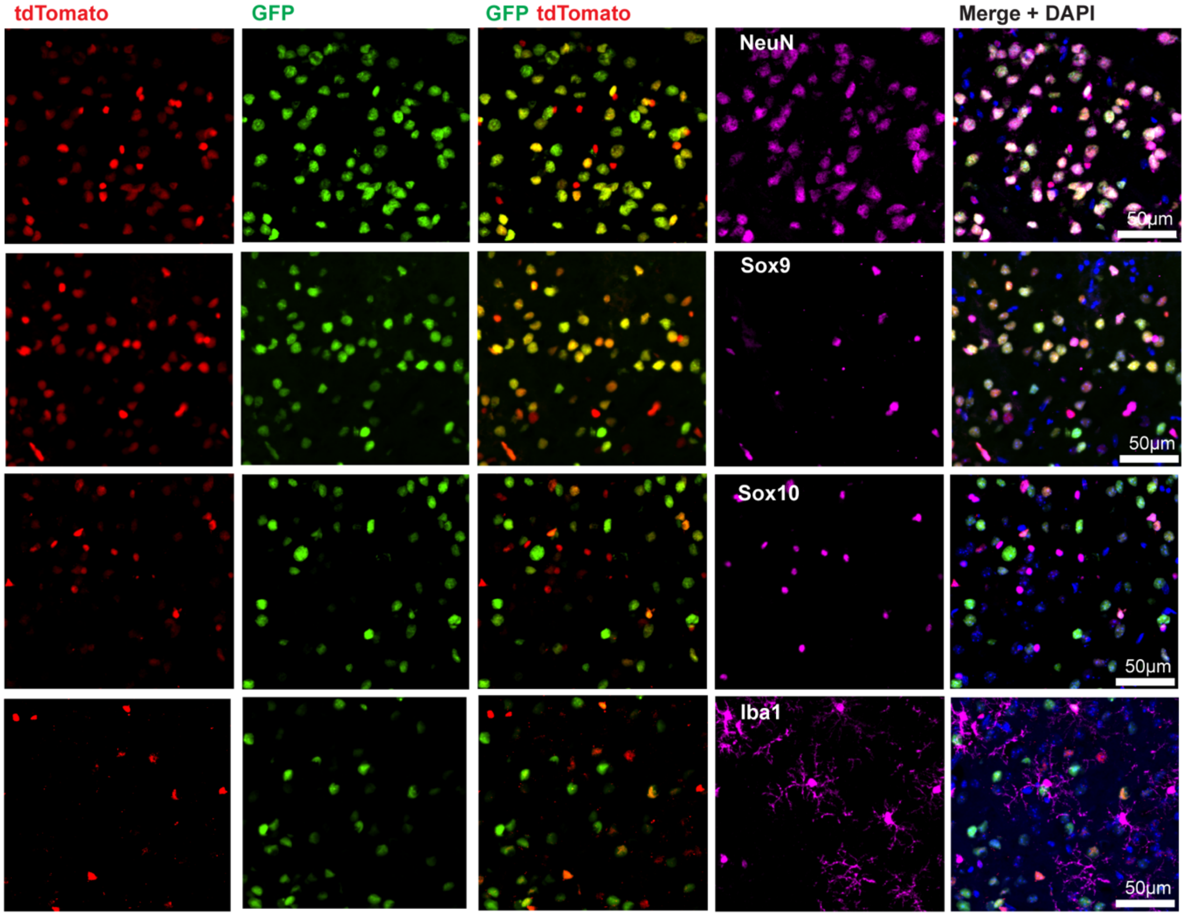
tdTomato
GFP
GFP tdTomato
Merge + DAPI
NeuN
Sox9
Sox10
Iba1
50μm

C

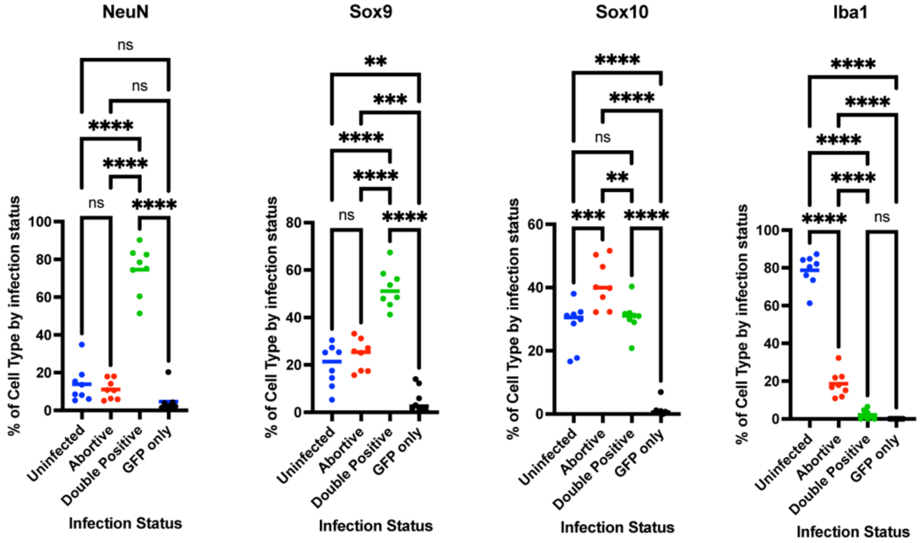
NeuN
Sox9
Sox10
Iba1
ns
****
**
***
% of Cell Type by infection status
Uninfected
Abortive
Double Positive
GFP only
Infection Status
0
20
40
60
80
100

Supplemental Figure 3: Larger area depicting cell types and infection status

A) Larger area of images from Figure 3. IHC of cell type-specific markers (magenta) depicted with infection markers, GFP and tdTomato (Cre recombination). B) Single channel panels of Figure 3 from main text are shown for depiction of weak signals. C) Graphs from Figure 3 but with productively infected cells (cells that contain GFP) separated by GFP+ only and GFP+ tdTomato+ (double positive). The graphs display means for each metric. Each point is from a separate animal (4 males, 4 females) with an average of 2-4 images per animal. One-way ANOVA was performed with Tukey's multiple comparison test for statistical analysis. $* \leq 0.0332$, $** \leq 0.0021$, $*** \leq 0.0002$, $**** \leq 0.0001$

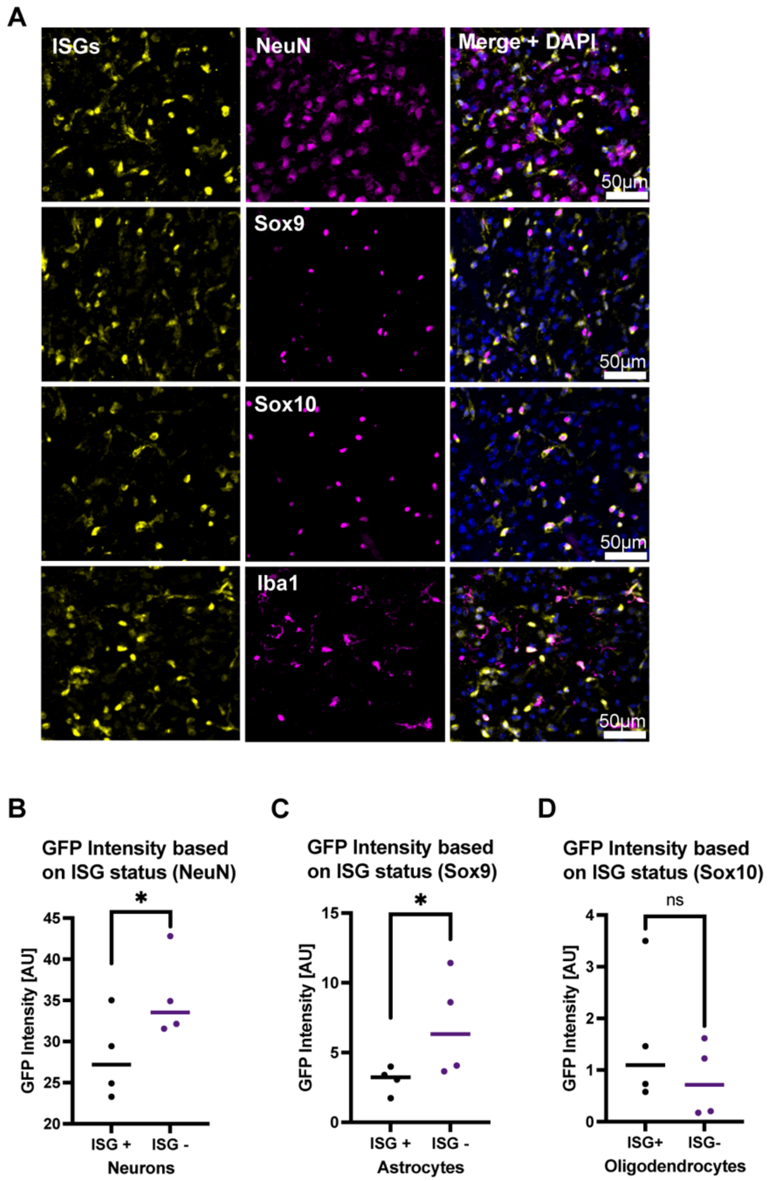

Supplemental Figure 4: GFP intensity in ISG+ versus ISG- cell populations by cell type

Pooled HCR-FISH probes detecting *ISG15, RSAD2,* and *IFIT3* were used to determine ISG expression and association with cell types in infected brains. A) Representative images of ISGs transcripts (yellow) and IHC for cell markers (magenta). Automated quantification of GFP intensity in cell types classified by association with ISGs. Graphs depicting GFP intensity across B) NeuN+, C) Sox9+, D) Sox10+ cells. Quantification of images taken 16hrs post infection. Graphs display means for each metric. Each point is from a separate animal (2 males, 2 females) with an average of 2-4 images per animal. One-way ANOVA was performed with Tukey's multiple comparison test for statistical analysis. $* \leq 0.0332$, $** \leq 0.0021$, $*** \leq 0.0002$, $**** \leq 0.0001$

A

	Grey Matter	White Matter
IFNa	-	-
IFNb	+	+
IFNl	-	-
IFNg	-	-

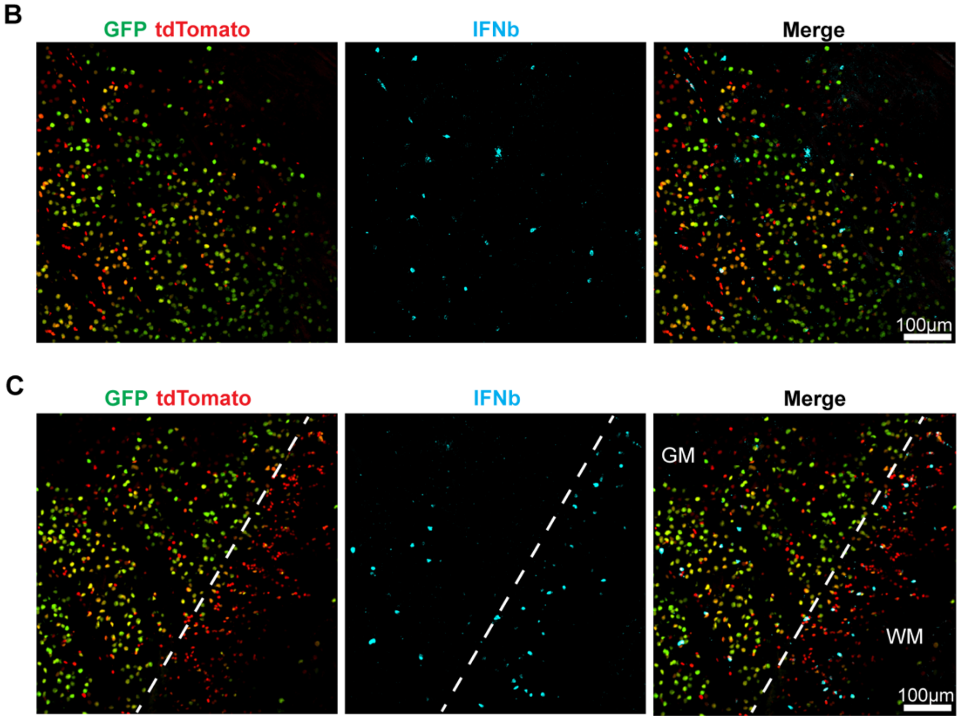

Supplemental Figure 5: Expression of IFN subtypes in DL-VSV infected brain

A) Table depicting HCR-FISH detection of IFN subtypes: *IFNa4, IFNb1*, *IFNl2, IFNg* (labeled IFNa, IFNb, IFNl, and IFNg) upon DL-VSV infection 16hrs post infection. Although probes were generated for *IFNa4, IFNb1*, *IFNl2, IFNg*; they cannot distinguish among different subtypes (e.g. IFNa4 vs IFNa2). B) IFNb detection in grey matter (striatum). C) IFNb detection in grey matter (GM) of striatum and white matter (WM) of corpus callosum. Images show GFP and tdTomato (Cre recombination readout) combined and IFNb separately along with a merge panel including all channels.

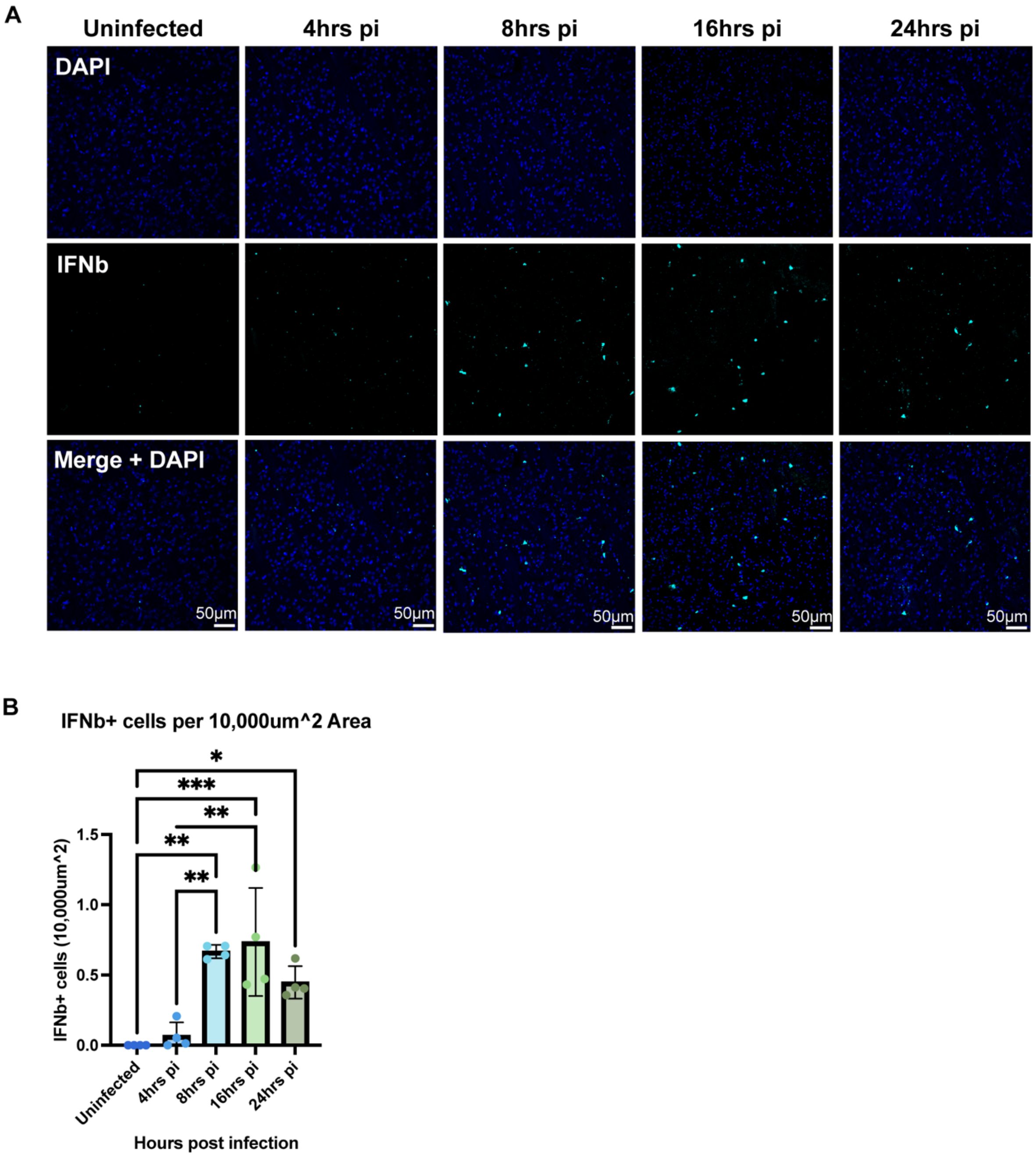

Supplemental Figure 6: *IFNb* expression time course post infection with DL-VSV

DL-VSV was injected into the striatum of Ai75d mice and the tissue was harvested at specific times post infection to determine onset of IFNb expression. A) Representative images showing DAPI staining along with HCR-FISH signal for *IFNb1* in an uninfected brain or brains harvested 4hrs, 8hrs, 16hrs, or 24hrs post infection. B) Quantification of IFNb induction time course. Nested One-way ANOVA was performed with Tukey's multiple comparison test for statistical analysis. $* \leq 0.0332$, $** \leq 0.0021$, $*** \leq 0.0002$, $**** \leq 0.0001$.

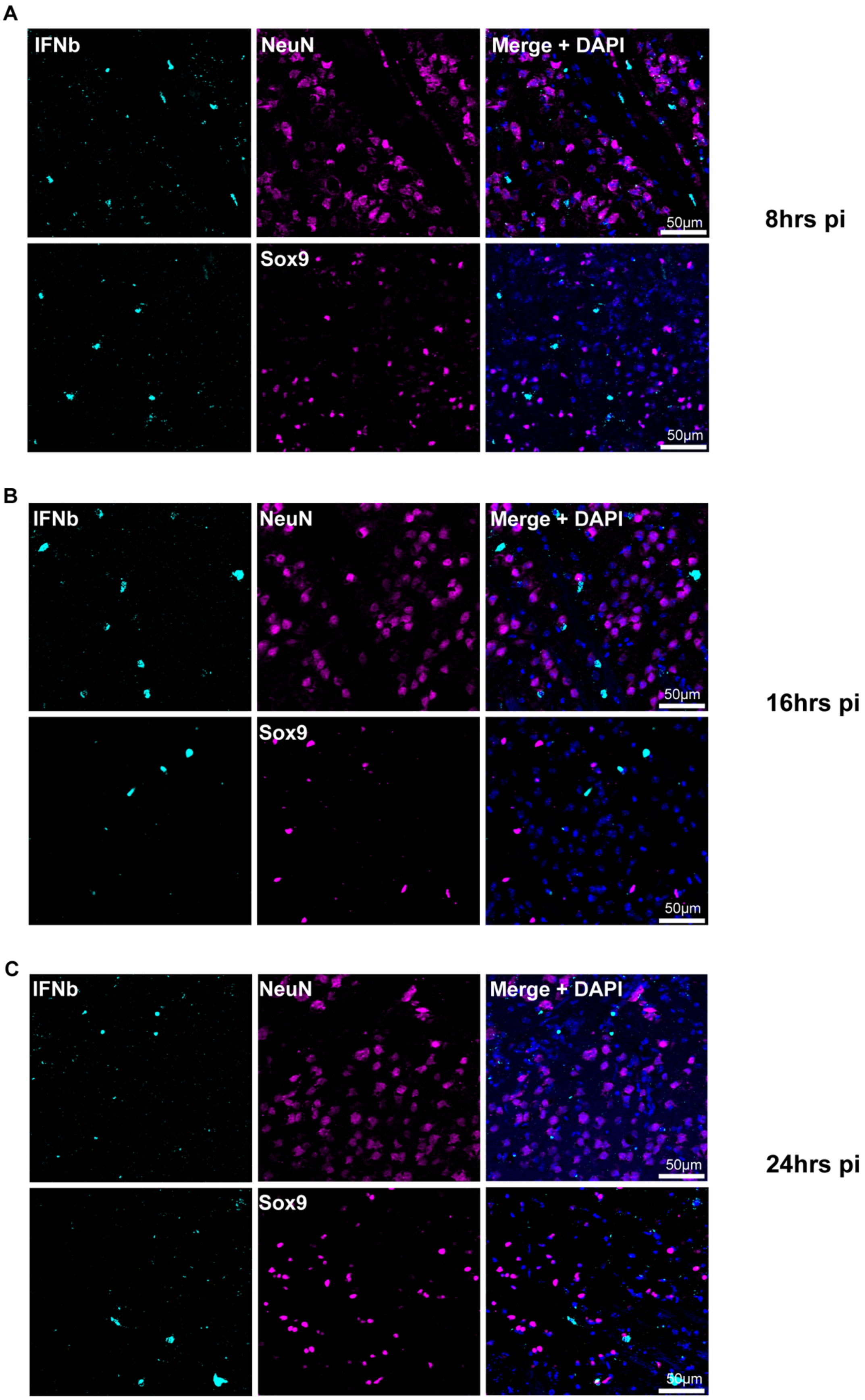
A
IFNb
NeuN
Merge + DAPI
50μm
Sox9
50μm
8hrs pi
B
IFNb
NeuN
Merge + DAPI
50μm
Sox9
50μm
16hrs pi
C
IFNb
NeuN
Merge + DAPI
50μm
Sox9
50μm
24hrs pi

Supplemental Figure 7: Expression of *IFNb* by NeuN+ and Sox9+ cells

Remaining images of cell types from Figure 6. HCR-FISH used to detect *IFNb* over a 24hr time course. Representative NeuN and Sox9 markers are shown with *IFNb* at A) 8hrs, B) 16hrs, and C) 24hrs post infection.

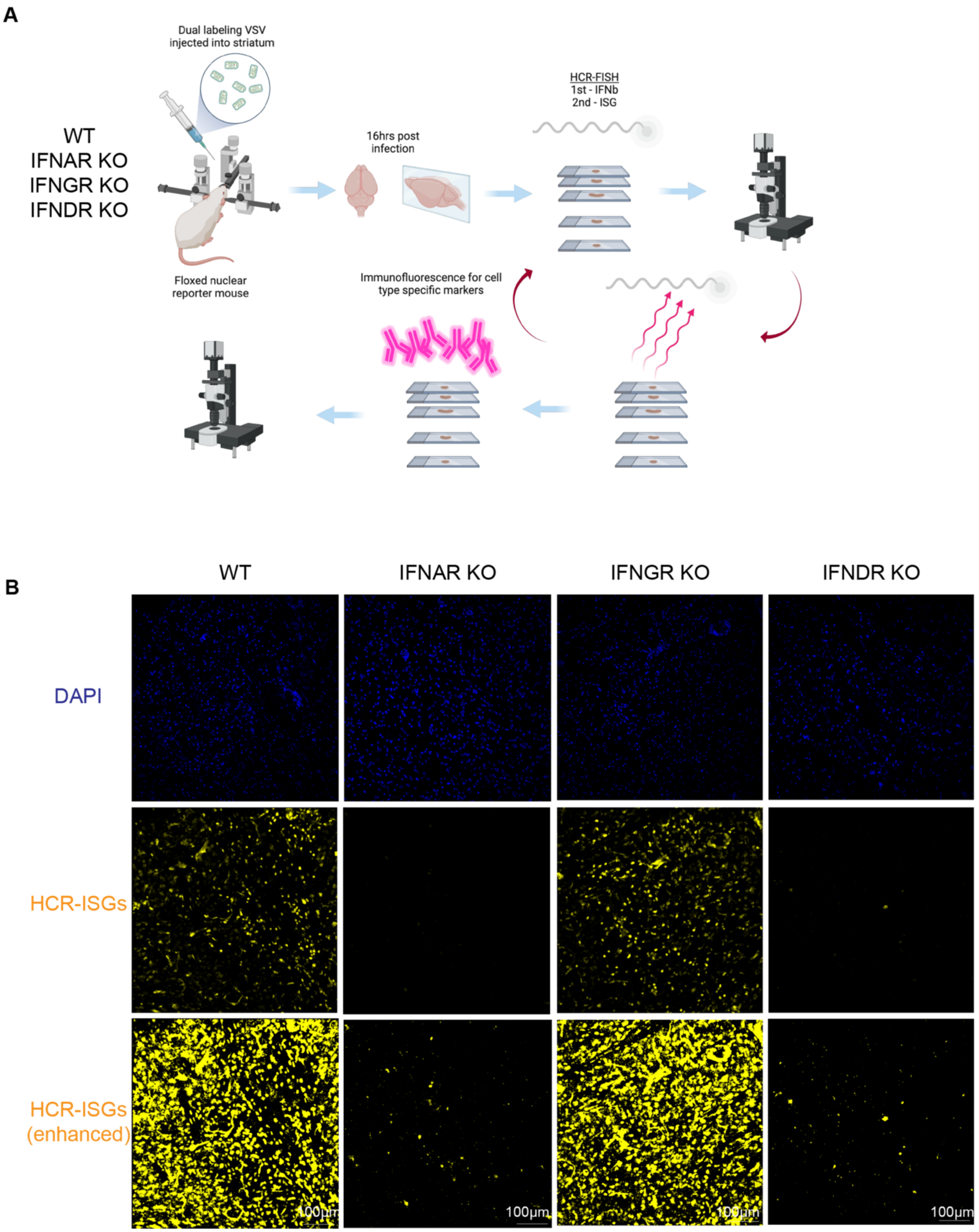
A
Dual labeling VSV
injected into striatum
WT
IFNAR KO
IFNGR KO
IFNDR KO
Floxed nuclear
reporter mouse
16hrs post
infection
HCR-FISH
1st - IFNb
2nd - ISG
Immunofluorescence for cell
type specific markers
B
WT
IFNAR KO
IFNGR KO
IFNDR KO
DAPI
HCR-ISGs
HCR-ISGs
(enhanced)
100μm
100μm
100μm
100μm

Supplemental Figure 8: IFN receptor KO effect on interferon stimulated genes (ISGs)

A) Experimental setup for addressing the role of IFN receptor signaling in DL-VSV primary infection. IFNAR KO, IFNGR KO, and IFN(A/G)R KO (i.e. IFNDR KO - lacking both IFNAR and IFNGR) were crossed with the Cre recombination reporter mice (Ai75d). Brains were harvested 16hrs post infection followed by serial HCR-FISH and IHC. B) HCR-FISH of ISGs (*ISG15, IFIT3, RSAD2)* (yellow) from infected brains in the separate IFN receptor KO mouse lines. Bottom row of panels shows enhanced brightness/contrast of ISG channel.

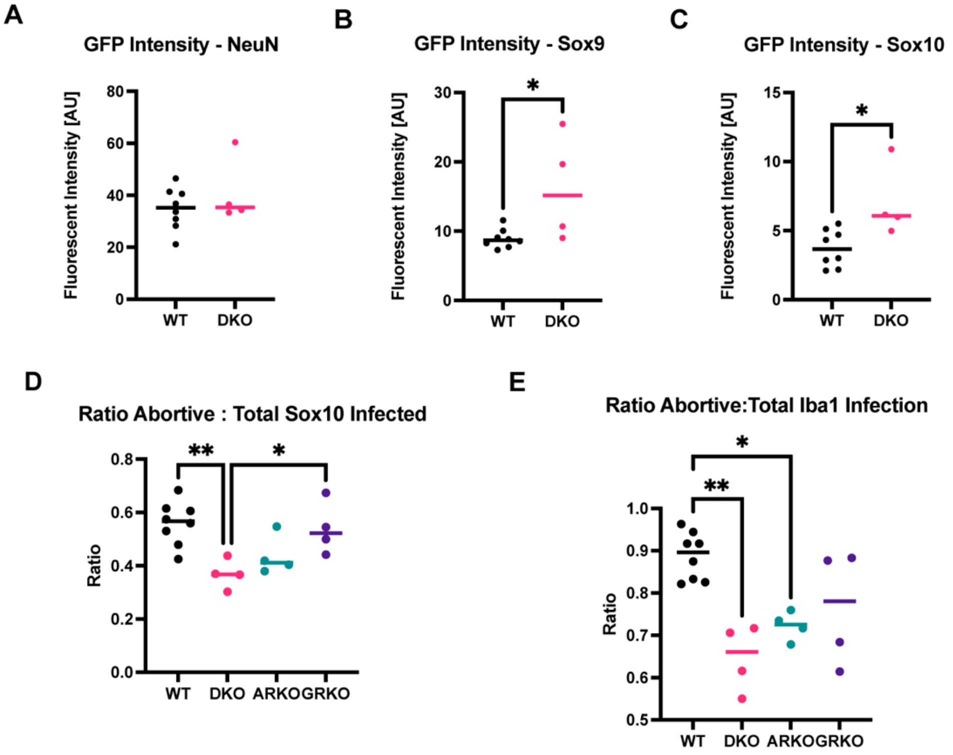

Supplemental Figure 9: Effect of IFNDR KO on GFP intensity in productively infected cells

Integrated intensity of GFP was compared between wt and IFNDR KO (labeled DKO) infected mice for A) NeuN+, B) Sox9+, C) Sox10+ cells. Infection ratios plotting abortive infection over total infection (abortive + productive) are plotted for D) Sox10 and E) Iba1 cells. Graphs display means for each metric. Each point represents averaged images from separate mice. Mice were divided evenly between males and females. Nested One-way ANOVA was performed with Tukey's multiple comparison test for statistical analysis. $* \leq 0.0332$, $** \leq 0.0021$, $*** \leq 0.0002$, $**** \leq 0.0001$
